# Generation of a caged lentiviral vector through an unnatural amino acid for photo-switchable transduction

**DOI:** 10.1093/nar/gkz659

**Published:** 2019-07-30

**Authors:** Yan Wang, Shuai Li, Zhenyu Tian, Jiaqi Sun, Shuobin Liang, Bo Zhang, Lu Bai, Yuanjie Zhang, Xueying Zhou, Sulong Xiao, Qiang Zhang, Lihe Zhang, Chuanling Zhang, Demin Zhou

**Affiliations:** 1 State Key Laboratory of Natural and Biomimetic Drugs, School of Pharmaceutical Sciences, Peking University, Beijing 100191, China; 2 Department of Molecular and Cellular Pharmacology, School of Pharmaceutical Sciences, Peking University, Beijing 100191, China; 3 Center for Translational Medicine, Chinese Academy of Medical Sciences and Peking Union Medical College, Beijing 100730, China; 4 Department of Pharmaceutics, School of Pharmaceutical Sciences, Peking University, Beijing 100191, China

## Abstract

Application of viral vectors in gene delivery is attracting widespread attention but is hampered by the absence of control over transduction, which may lead to non-selective transduction with adverse side effects. To overcome some of these limitations, we proposed an unnatural amino acid aided caging–uncaging strategy for controlling the transduction capability of a viral vector. In this proof-of-principle study, we first expanded the genetic code of the lentiviral vector to incorporate an azido-containing unnatural amino acid (Nϵ-2-azidoethyloxycarbonyl-l-lysine, NAEK) site specifically within a lentiviral envelope protein. Screening of the resultant vectors indicated that NAEK incorporation at Y77 and Y116 was capable of inactivating viral transduction upon click conjugation with a photo-cleavable chemical molecule (T1). Exposure of the chimeric viral vector (Y77-T1) to UVA light subsequently removed the photo-caging group and restored the transduction capability of lentiviral vector both *in vitro* and *in vivo*. Our results indicate that the use of the photo-uncage activation procedure can reverse deactivated lentiviral vectors and thus enable regulation of viral transduction in a switchable manner. The methods presented here may be a general approach for generating various switchable vectors that respond to different stimulations and adapt to different viral vectors.

## INTRODUCTION

Gene therapy, a clinical treatment that introduces a therapeutic gene into a cell to replace or repair an abnormal gene and eradicate the aberration (1), mainly consists of two basic elements: a therapeutic nucleic acid and a nucleic acid carrier. The development of RNA interference and CRISPR gene editing heralded a major advance in nucleic acids therapy that could be used in humans (2,3). Viral vectors, mainly derived from adeno-associated virus and lentiviruses, have found an encouraging new beginning in gene therapy (4–8). A desirable feature for a viral vector is the natural advantage as a transporter to achieve direct, targeted delivery of a gene of interest with prolonged gene expression (1,9,10). However, the unmanageable characteristics of viral transduction, which lead to non-selective delivery and safety issues, have hampered the broad applications of viral vectors in both basic and translation studies (11). Making viral vectors as controllable as possible remains a topic of ongoing investigation (12–20). The potential improvements in vector engineering may place viral vector-based therapy at the forefront of modern medicine to treat various diseases (21,22). Lentiviral vectors, composed of a vesicular stomatitis virus envelope glycoprotein (VSVg) insert plus the enzymes and RNA genome of Human Immunodeficiency Virus serotype 1 (23), have received particular preference among many different vectors due to their ability to transduce both dividing and non-dividing cells and integrate the foreign gene into the host genome, thereby producing stable transgene expression (24–28). The random integration of lentiviral vectors can potentially lead to insertional mutagenesis with unacceptable carcinogenic potential (23,29). Various methods have been attempted to achieve site-specific integration or block integration of retroviral DNA to overcome this limitation (30–32). Alternatively, making the viral transduction as controllable as possible is another way to alleviate some of the safety concerns caused by the random integration.

The component of lentiviral vectors that determines delivery efficiency is the envelope VSVg whose interactions mediate virus-cell fusion, internalization, and transport in host cells (33,34). We previously established a novel strategy for expanding the genetic code of the lentiviral vector, whereby unnatural amino acids (UAAs) containing azido (Nϵ-2-azidoethyloxycarbonyl-l-lysine, NAEK) were site-specifically incorporated on the surface of the lentivirus (35). High-fidelity expansion of the genetic code plus facile mutagenesis allows precise placement of azide-containing UAAs following conjugation with certain ligands. These provide a foundation for the systematic exploration of perturbations in ligand-guided function such as localizing sites of transduction blockage by alteration of the ligand locus and associated vector structure. UAA-tag positions on the envelope of the lentiviral vector were identified that had minimal effect on either production or transduction. In addition to the targeted sites that were modified, screening of the resultant vectors revealed certain other sites on the lentiviral vector that were tolerant to UAA incorporation but, following conjugation with ligands, did not allow transduction of the modified viral vector (35).

Inspired by our previous findings, we proposed an UAA aided caging-uncaging strategy that causes the viral vector to transduce genes only within target tissues, thereby overcoming some of the limitations of uncontrollable transduction. To briefly review, a viral vector is temporarily inactivated by an UAA mediated site-specific modification and can restore its transduction capacity by removing this modification via stimulation. In this proof-of-principle study, we report the generation of a photo-cleavable molecule caged lentiviral vector whose transduction can be controlled by ultraviolet radiation in a photo-switchable manner. We believe this new vector construct shows promising potential in applications for gene therapy and basic scientific research. The methods presented here may become precursor for a general approach for generating various switchable vectors that respond to different stimulations, such as pH and hypoxia that can be adapted to different viral vectors.

## MATERIALS AND METHODS

### Plasmids, antibodies and reagents

Plasmids for the production of the lentiviral vector pCMV-VSVG, pNL 4–3 and pAdvantage were obtained from Promega (Sunnyvale, CA, USA). Mutant plasmid (pCMV-VSVG-TAG) harboring an amber codon within the *VSVG* gene was generated from wild-type pCMV-VSVG using the QuikChange Lightning Site-Directed Mutagenesis kit (Agilent Technologies, Santa Clara, CA, USA). A plasmid containing pyrrolysyl tRNA synthetase/tRNA_CUA_ pair (PylRS/tRNA_CUA_) for site-specific incorporation of azido-containing unnatural amino acid (Nϵ-2-azidoethyloxycarbonyl-l-lysine, NAEK) was reported in our previous paper (35). All plasmids used for transfection were purified with a Maxiprep kit (Promega).

Mouse anti-VSVg monoclonal antibody was obtained from Sigma-Aldrich (V5507, St Louis, MO, USA). Rabbit monoclonal anti-glyceraldehyde 3-phosphate dehydrogenase antibody was purchased from Cell Signaling Technology (Beverly, MA, USA). Horseradish peroxidase-conjugated goat anti-mouse and anti-rabbit IgG were obtained from ZSGB-BIO (Beijing, China). Alexa 488-labeled anti-mouse IgG and rabbit anti-Giantin antibody (a Golgi marker) were purchased from Abcam, USA (ab150117 and ab80864, respectively). Alexa 488-labeled anti-rabbit IgG (#4412) and Alexa 555-labeled anti-mouse IgG were obtained from Cell Signaling Technology. NAEK was synthesized as previously reported (35). Dibenzocyclooctyne (DIBO)-RGD was purchased from GL Biochem (Shanghai, China). DIBO-Alexa 488/555 and DAPI were obtained from Life Technologies (Carlsbad, CA, USA). DIBO-folic acid (DIBO-FA) and the photo-cleavable molecules (T0, T1 and T2) were synthesized in our laboratory; the structures and synthetic protocols can be found in the Supplementary Data.

### Cell culture

Human embryonic kidney 293T (HEK-293T) and HeLa cells were obtained from the National Platform of Experimental Cell Resources for SCI-Tech (Beijing, China). Michigan Cancer Foundation (MCF)-7 was a gift from Professor Hongquan Zhang of Peking University School of Basic Medical Sciences (Beijing, China). KB cells were kindly provided by Prof. Xinjing Tang of Peking University. HEK-293T, HeLa and MCF-7 cells were maintained in Dulbecco's modified Eagle's medium (DMEM) from Macgene (Beijing, China). KB cells were maintained in 1640 medium from Gibco (Grand Island, NY, USA). All cultures were supplemented with 10% (v/v) fetal bovine serum (FBS; Gibco), 100 IU/ml penicillin and 100 μg/ml streptomycin (Macgene). The cells were cultured at 37°C in a humidified incubator with 5% CO_2_ and were passaged every 3 days when they reached 80–90% confluence.

### Propagation of wild-type and NAEK-labeled lentiviral vectors

The detailed procedure for the propagation of wild-type lentiviral vectors was previously described (35). The same procedure was followed to produce NAEK-labeled lentiviral vectors, except that the pCMV-VSVG plasmid was replaced with the mutant pCMV-VSVG-TAG and aaRS/tRNA (PylRS/tRNA_CUA_) plasmids (0.35 μg) and the medium was supplemented with 1 mM NAEK.

### Viral titer test

To determine the functional titer of lentivirus, HEK293T, HeLa cells (7 × 10^3^ per well) and MCF-7 cells (1.5 × 10^4^ per well) were seeded in a 96-well plate. The following day, a virus sample was added in triplicate to the 96-well plate. After 24 h, the culture medium was replaced with fresh medium followed by incubation for an additional 48 h. The cells were then analyzed for transduction. Transduction capability of the vector harboring the luciferase gene was determined using the Bright-Glo Luciferase Assay System (Promega), and that of the vector containing the GFP gene was determined by flow cytometry. The genomic titer of lentiviral particles was determined by Lenti-Pac HIV™ qRT-PCR Titration Kits (GenoCoposia, Beijing, China) following the manufacture's protocol. All quantitative data were expressed as average values with standard deviations from triplicate experiments.

### Conjugation of NAEK- lentiviral vectors conjugated to Alexa 488, RGD ligand, FA ligand and photo-cleavable molecules

Folic acid (FA) ligand was conjugated to the NAEK-labeled lentiviral vector by a standard procedure that has been used for conjugation of Alexa 488/555 and RGD ligand (35). Alexa 488-conjugated lentiviral vectors (WT, Y77 and Y116) were mixed with loading buffer, boiled for 10 min and separated by sodium dodecyl sulfate polyacrylamide gel electrophoresis (SDS-PAGE) on a 12% gel and visualized by fluorescence imaging and Coomassie Blue staining. NAEK-labeled lentiviral vector was conjugated to a photo-cleavable molecule as follows. The Y77 or Y116 NAEK mutant lentiviral vector was incubated overnight at 4°C with either T0, T1 or T2 (at 100 μM each). The conjugated products were purified by ultrafiltration using a 100 kD membrane filter and phosphate-buffered saline (PBS) as a buffer to remove unreacted T0/T1/T2-DIBO. The T0/T1/T2-conjugated lentiviral vectors were then used for testing viral transduction.

### Transduction and integration of RGD/T1-modified lentiviral vectors

To determine *in vitro* transduction behavior of the RGD/T1-modified lentiviral vectors, HEK293T, HeLa or KB cells were seeded in a 96-well optical bottom plate (NUNC #165305; Thermo Fisher Scientific) at ∼1 × 10^4^ cells per well. Twenty hours afterward, the cells were transduced with 10 μl (∼5 × 10∧8 genomic copies/ml) of concentrated RGD/T1-conjugated or wild-type lentiviral vector containing a luciferase or GFP reporter gene. Fluorescence images were acquired with a fluorescence microscope (Nikon, Tokyo, Japan) at 48, 72, 96 and 120 h after transduction. Nuclei were stained with DAPI (blue). Luciferase activity was tested 2, 3, 5, 7 and 9 days after transduction using the Bright-Glo Luciferase Assay System (Promega, Sunnyvale, CA, USA).

The integration of the *GFP*-harboring lentiviral vector was verified by polymerase chain reaction (PCR) using the total genomic DNA as the template. Total genomic DNA of the HEK293T cells transduced with lentiviral vectors for 120 h was extracted by the Wizard Genomic DNA Purification kit (Promega A1120, MD, USA). The PCR contained 10 μl Go Taq Green Master Mix (Promega M7121), 1 μl of genomic DNA and 0.5 pM each of the forward primer GFP-F (5′-CTTCTTCAAGTCCGCCATGC-3′) and reverse primer GFP-R (5′-CTTCTCGTTGGGGTCTTTGC-3′). The negative control consisted of templates of genomic DNA of HEK293T cells without transduction, nuclease-free water, and wild-type lentivirus containing GFP as reporter. The cycling conditions were as follows: 5 min at 94°C; 30 cycles of 30 s at 94°C, 30 s at 58°C and 30 s at 72°C; and a final extension for 10 min at 72°C. PCR fragments were resolved by agarose electrophoresis on a 1% agarose/Tris-acetate-ethylenediaminetetraacetic acid (TAE) gel stained with GelRed (Biotium 41003–0.5 ml).

### Immunofluorescence analysis of lentiviral vector-transduced cells

HeLa cells were seeded on 35 mm glass-bottom culture dishes 24 h before immune-labeling. When they reached 40–70% confluence, cells were incubated with 15 μl (∼5 × 10∧8 genomic copies/ml) unmodified or modified lentiviral vectors at 4°C for 1 h to synchronize viral transduction. After removing excess lentiviral vectors, transduced cells were fixed with 4% paraformaldehyde for 15 min at room temperature. Other dishes were fixed with paraformaldehyde after 1, 3, 6 or 24 h followed by incubation at 37°C in a humidified incubator. The cells were permeabilized by 0.2% (v/v) Triton X-100 in PBS for 15 min at room temperature. The treated cells were blocked with buffer composed of 5% (v/v) FBS in PBS for 1 h at room temperature and sequentially incubated with 2 μg/ml mouse anti-VSVg antibody overnight at 4°C followed by 1 μg/ml Alexa Fluor 488 goat anti-mouse IgG (Abcam, Cambridge, USA) for 1 h in the dark, and then stained with 2 drops/ml DAPI to label the nuclei (Life Technologies) for 20 min in the dark at room temperature. For co-localization analysis of the lentivirus in the Golgi apparatus, the samples were incubated with 2 μg/ml mouse anti-VSVg and 1 μg/ml rabbit anti-Giantin antibody overnight at 4°C at the same time, followed by incubation with 1 μg/ml Alexa Fluor 555-labeled goat anti-mouse IgG (CST) and 1 μg/ml Alexa Fluor 488-labeled goat anti-rabbit IgG (CST) for 1 h in the dark. The samples were rinsed three times with 0.1% (v/v) Tween-20 in PBS between each step. Cells were imaged using a confocal laser-scanning microscope (Nikon, Tokyo, Japan).

### Quantitative analysis of the photo-cleavage efficiency of T0, T1 and T2

T0 (or T1, T2) was diluted with an acetonitrile solution of 10% dimethyl sulfoxide (DMSO) to a final concentration of 5 mM. The solution (100 μl per Eppendorf (EP) tube) was irradiated using a UV-LED lamp (365 nm, 7 mW/cm^2^) for 0, 1, 2, 3, 5, 8 and 10 min, and the photo-cleavage yields of the samples were analyzed by high-pressure liquid chromatography (HPLC). Analytical HPLC was performed on an Agilent 1260 separation module connected to a G1314F VWD detector using a Zorbax Eclipse Plus C18 HPLC column (100 × 4.6 mm, 3.5 μm). Five microliters of each sample were injected into the HPLC column and elution was carried out with a flow rate of 1.0 ml/min while the column temperature was maintained at 25°C. The mobile phase was composed of A (water) and B (acetonitrile solution) with the following gradient elution protocol: 0 min, 5% B; 0–5 min, 5–60% B; 5–8 min, 60–70% B; 8–10 min, 70–90% B; 10–13 min, 90–95% B; 13–16 min, 95% B; 16–20 min, 95–5% B. The decaging efficiency (DE%) of T0 (or T1 or T2) = n(min) peak area/0(min) peak area. The 365 nm ultraviolet absorption efficiencies of T0, T1 and T3 are more than 99.9%; thus, we posit that the photons absorbed equal the emission photons. The quantum yield was calculated using the following formula:

$$\begin{eqnarray*}&& {\rm{Quantum\ Yield\ }}\left( {{\rm{QY}}} \right) = \frac{{\# {\rm molecules}{\rm{\ }}{\rm decomposed}}}{{\# {\rm photons}{\rm{\ }}{\rm {\rm absorbed}}}} \nonumber\\ &&\quad = \frac{{n\left( {T0{\rm{\ }}or{\rm{\ }}T1{\rm{\ }}or{\rm{\ }}T2} \right)}}{N} = \frac{{C \times V \times {\rm{\ NA\ }} \times DE{\rm{\% }}}}{{\frac{{P{\rm{\lambda }}}}{{hc}} \times t}} \nonumber\\ &&\quad = \frac{{5 \times {{10}^{ - 3}} \times 50 \times {{10}^{ - 6}} \times 6.02 \times {{10}^{23}} \times DE{\rm{\% }}}}{{\frac{{7 \times {{10}^{ - 3}} \times 365 \times {{10}^{ - 9}}}}{{6.63 \times {{10}^{ - 34}} \times 3 \times {{10}^8}}} \times t}} \nonumber\\ &&\quad = {\rm{\ }}11.716{\rm{\ }} \times \frac{{DE{\rm{\% }}}}{t}\end{eqnarray*}$$

where C is the Molar concentration (mol/L), V is the volume (L), NA is Avogadro's constant (6.02 × 10^23^/mol), P is the power of the UV-LED lamp (W), λ is the wavelength (m), h is Plank's constant (6.63 × 10–34 Js), c is the speed of light (m/s) and t is the radiation time (s).

### Bioluminescence imaging of living cells

For photocleavage experiments and time-dependent live-cell imaging, HEK293T cells were seeded in a 96-well optical bottom plate (NUNC #165305; Thermo Fisher Scientific) at ∼1 × 10^4^ cells per well at 20 h before introduction of 50 μl (∼5 × 10∧8 genomic copies/ml) T0/T1/T2-conjugated lentiviral vectors (with luciferase or GFP as the reporter gene). After changing the culture medium, the cells were exposed to UV radiation for 1–10 min (365 nm, 7 mW/cm^2^). At 72 h after transduction, the culture medium was removed and 50-μl of Bright-Glo reagent (Promega) was added to each well, and the luminescence was detected with an illuminometer (Centro XS3 LB 961; Berthold Technologies, Bad Wildbad, Germany). For GFP-lentivirus, GFP fluorescence was detected on an inverted fluorescence microscope, and GFP-positive cells were quantified by flow cytometry.

### *In vivo* test of the photo-switchable lentiviral vector

Female BALB/C nude mice (nude mice), 6–8 weeks of age, were purchased from Beijing Vital River Laboratory Animal Technology Co., Ltd. and maintained under pathogen-free conditions in-house on a 12-h light cycle with access to food and water *ad libitum*. All protocols were approved by the Institutional Animal Care and Use Committee. For the UV cleavage assay, a subcutaneous transplantation tumor model was established. On day 0, nude mice were inoculated subcutaneously with 3 × 10^6^ KB cells on the right flank. Tumors became palpable after about 5 days (50-100 mm^3^). The mice were then intratumorally (I.T.) injected with 200 μl (∼5 × 10∧8 genomic copies/ml) of concentrated Y77 (positive control, group #1, Figure 7B) or Y77-T1 virus (groups #2 & 3, Figure 7B). After Y77-T1 virus injection, the mice in group 3 were subjected to 365 nm UV irradiation through the skin for 5 min using a 3W flashlight (UVAA02, Tank007, Beijing, China). In parallel, negative control groups were injected with Y77-T1 (group #2, Figure 7B) only, absent irradiation. There were six mice per group. Transduction of the viral vector containing luciferase as a reporter gene was detected 4 days after UV irradiation by bioluminescence imaging.

### Statistical analysis

Two-paired Student's *t*-tests were used to calculate *P-*values. Differences with *P-*values < 0.05 were considered statistically significant. All graphs and test statistics were generated using Graphpad Prism 6.0.

## RESULTS

### Site-specific modification of lentiviral vector by incorporation of unnatural amino acid

In our previous study, we developed a method for site-specific modification of viral vector based on genetic code expansion, which included incorporation of an unnatural amino acid containing an azido group (NAEK) in a viral membrane protein and subsequent conjugation of targeting molecules via a click chemistry reaction (35). In the present study, 33 residues of the VSVg protein located in the protein recognition, fusion and trimer-binding domains and on the VSVg protein surface (33) were selected for NAEK incorporation. A total of 33 NAEK-incorporated lentiviral vectors were produced, and their functional titers were evaluated by luciferase assay and normalized to that of the wild-type lentiviral vector (Supplementary Figure S1A). In total, 26 NAEK-modified lentiviral vectors showed distinct transduction capability. Vectors modified with NAEK at residues Y73, K200, Y281, W288, R332, D334 and G345, did not transduce the cells, possibly due to the unsuitability of these residues for NAEK incorporation according to our previous study (35). To examine the structure-activity relationship of the NAEK-modified lentiviral vector, the genomic titers and the expression levels of VSVg that reflect the ability to incorporate NAEK at the defined positions Y77, Y116, D192 and K225 were separately tested by real-time RT-PCR and Western blotting (Supplementary Figure S1B and C). The capacity for viral transduction was measured by the ratios of the functional titers to the genomic titers, with the aim to clarify whether the incorporations of NAEKs among these positions affect viral transduction capability. As shown in Supplementary Figure S1D, both the transduction capability and protein expression of VSVg resulting from mutations at positions Y77, Y116 and D192 showed different levels of reduction compared to that of wild-type lentiviral vectors, indicating that the incorporation of NAEKs at these positions resulted in a loss of viral transduction capability. However, the mutant at position K225 showed a slight increase in viral transduction capability compared to that of the wild-type lentiviral vector. These results suggested that the lentiviral vector can tolerate the addition of NAEK to varying degrees at different sites.

**Figure 1.**
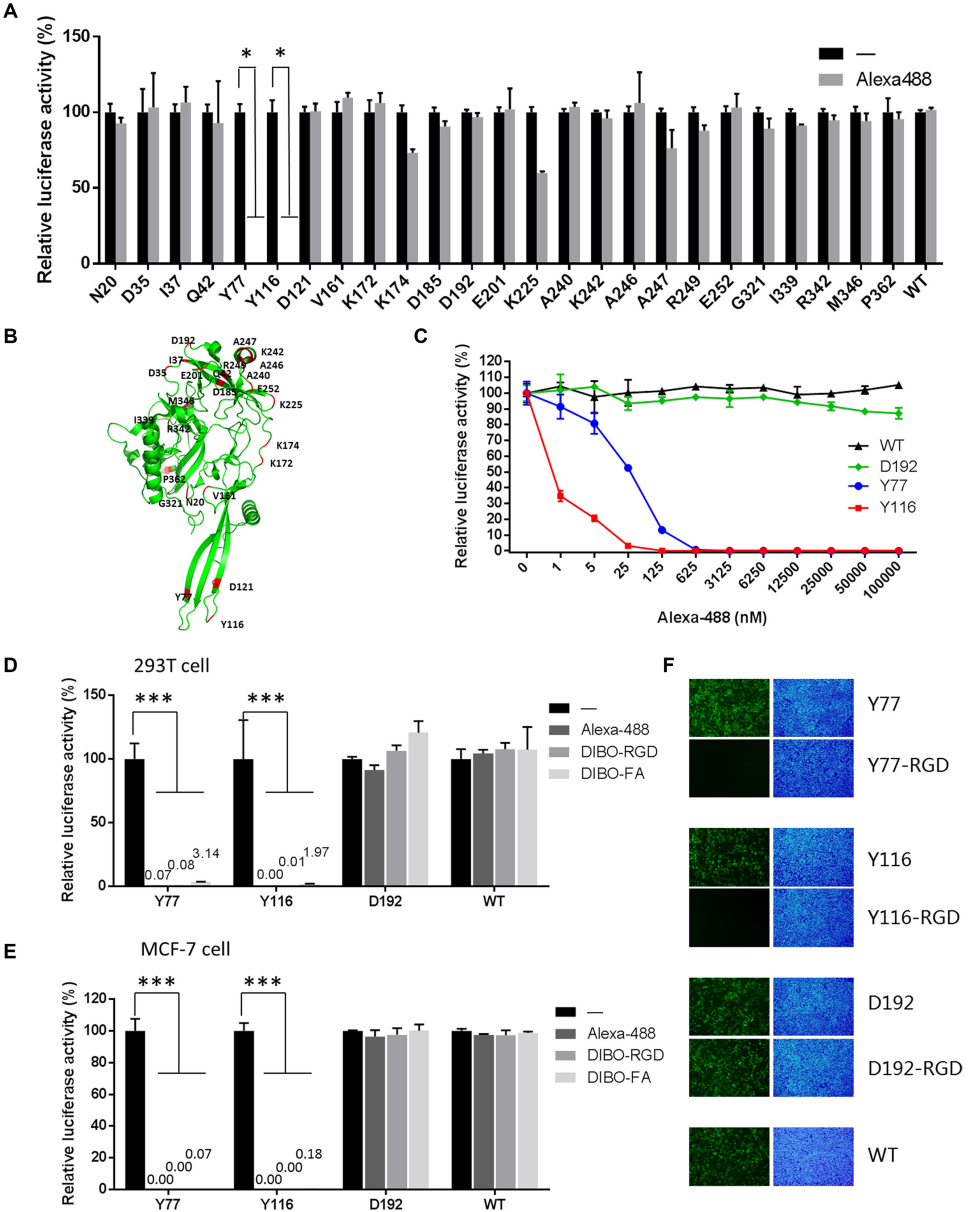
Screen for transduction capability of NAEK substituted lentiviruses modified at various blocking residues. (**A**) Effect of modification by Alexa Fluor-488 at different sites of pseudotyped VSVg on viral transduction capability, as measured by relative luciferase activity. *P < 0.05 versus corresponding control, *n* = 3. (**B**) Schematic illustration of unnatural amino acid insertion sites on VSVg. (**C**) Effect of different concentrations of Alexa Fluor-488 on the transduction capability of lentiviral vectors modified with NAEK at residues Y77, Y116 and D192, as measured by relative luciferase activity. (**D** and **E**) Quantitative analysis of the effects of Alexa Fluor-488, cRGD and FA modifications on viral transduction ability in 293T cells (D) and MCF7 cells (E). ***P < 0.001, **P < 0.01 versus corresponding control, *n* = 3. (**F**), Fluorescence micrographs of 293T cells transduced with RGD-modified lentiviral vectors harboring the GFP reporter gene. Green fluorescence identifies virus-transduced cells; nuclei stained with DAPI appear blue. ‘-’ indicates no conjugation as a control.

To evaluate the compatibility of NAEK on the viral surface to undergo click chemistry and to check the effect of NAEK conjugation on the capacity for viral transduction, NAEK and Alexa Flour-488 modified vectors were evaluated for their transduction efficacy based on relative luciferase activity. The results suggest that transduction was blocked in a dose-dependent manner with lentiviral vectors modified with Alexa Fluor-488 at residues Y77 and Y116 (i.e. Y77- and Y116-Alexa Fluor-488, respectively) (Figure 1A–C). The covalent conjugation of Y77- and Y116-Alexa Fluor-488 was confirmed by SDS-PAGE and fluorescence imaging (Supplementary Figure S1E). Given that Alexa-488 conjugation can block viral transduction, we investigated whether other molecules such as RGD and FA would also have this effect and if it is a common outcome in different cell types. The transduction capability of the DIBO-Alexa Fluor-488, -RGD, and -FA modified vectors were evaluated based on the relative luciferase activity exhibited in 293T (Figure 1D), MCF-7 (Figure 1E) and HeLa cells (Supplementary Figure S1F). All of the conjugated molecules blocked lentiviral transduction when the modification was at residues Y77 and Y116. On the other hand, no blocking was detected with the wild-type (control) and D192 viral vectors. Similar results were also obtained from the fluorescent images visually indicating the success of viral transduction (Figure 1F and Supplementary Figure S2). These results indicate that any conjugation at Y77and Y116 is able to block viral transduction in a range of cell types including both normal and tumor cells.

### Caging a lentiviral vector in its inert state via UAA linked at VSVgY77 or Y116

**Figure 2.**
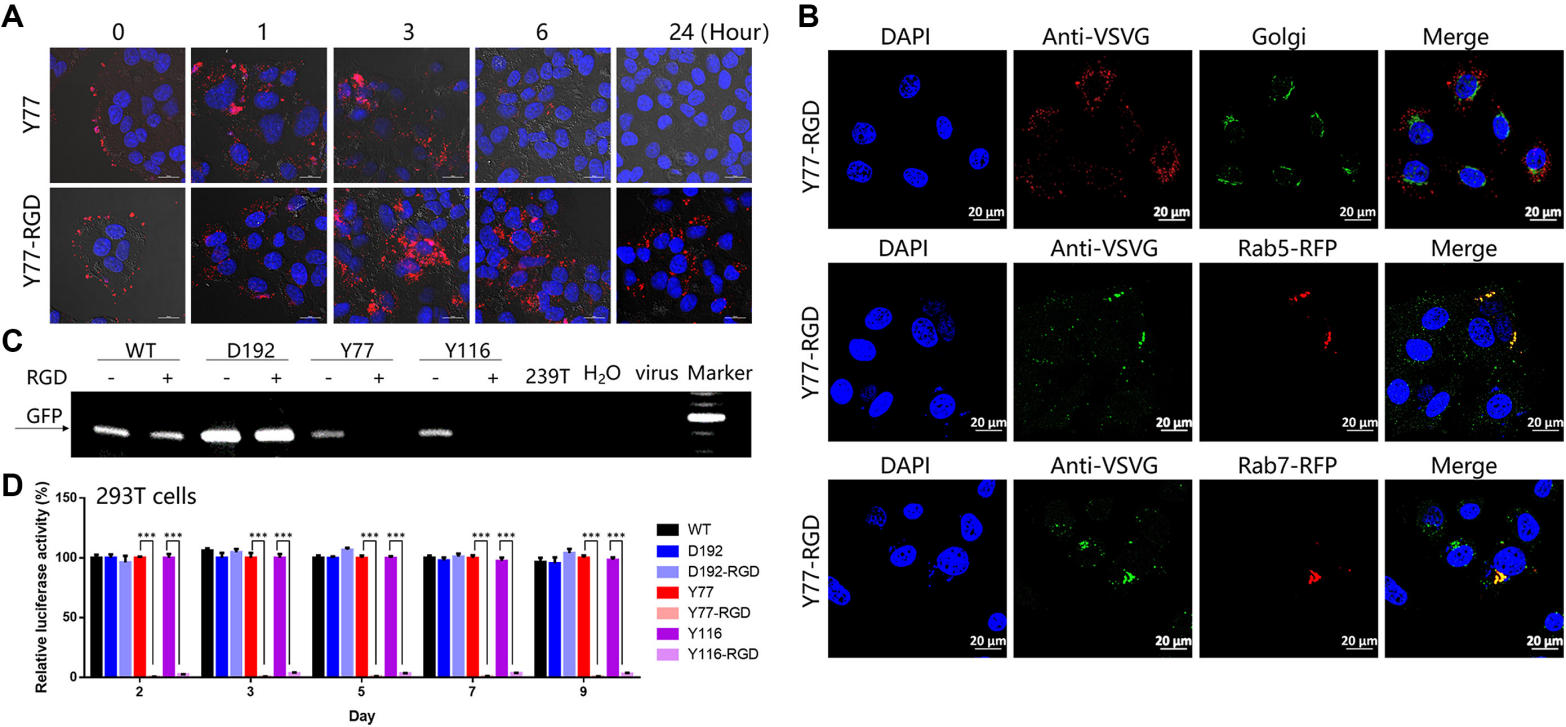
Transduction pathway and integration competence of the RGD-modified lentiviral vector. (**A**) Subcellular localization analysis of Y77-RGD vector (red) by immunofluorescence labeling in HeLa cells. Scale bar, 20 μm. (**B**) Co-localization analysis of Y77-RGD (green) and Rab5/7-red fluorescent protein (RFP) (red) as markers of early/late endosomes, or Y77-RGD (red) and Golgi (green) in HeLa cells 6 h after viral transduction. Nuclei were stained with DAPI (blue). Scale bar, 20 μm. (**C**) Integration capability of the RGD-modified lentiviral vector analyzed by PCR amplification of *GFP* sequences using genomic DNA as templates. The genomic DNA was extracted from 293T cells transduced with wild-type or D192/Y77/Y116 ± RGD vectors. Genomic DNA extracted from blank 293T cells and a template using water or lentivirus were used as negative controls. PCR products were evaluated on a 1% agarose gel stained with GelRed. Marker, 1-kb ladder. (**D**) Quantitative analysis of the effects of cRGD modifications on viral transduction ability in 293T cells. The luciferase activity was tested 2, 3, 5, 7 and 9 days after transduction (***P < 0.001, *n* = 3).

To investigate the blocking mechanism of Y77- and Y116-RGD viral vectors, we analyzed the crystal structure of VSVg. The Y77 and Y116 residues are both located in the VSVg membrane-fusion domain that plays a key role in mediating the viral envelope, membrane fusion and viral release from the endosome into the cytoplasm (33,34). We speculated that modification of these two sites hinders the membrane fusion process, resulting in loss of viral transduction capability. To test this hypothesis, we observed the localization of viral particles in the cells by immunofluorescence detection and confocal microscopy. Irrespective of the specific modification, the fluorescence of the Y77 group diminished after 6 h, indicating that most of the viral particles had completed the transduction phase and the VSVg protein had been degraded by the cells. The Y77-RGD group still showed obvious fluorescence in cells 24 h after transduction (Figure 2A). A similar phenomenon was observed with the Y116-RGD virus (Supplementary Figure S3A). These results indicate that Y77-RGD and Y116-RGD viruses were blocked in the cytoplasm after entry into the host cell.

The spread of punctate tags throughout the cytoplasm suggested that the viral vectors may be blocked in the Golgi or endosome. To investigate whether RGD-conjugated vectors were trapped in the Golgi, the Golgi of HeLa cells transduced with Y77-RGD or Y116-RGD were immuno-stained and then analyzed by confocal microscopy. We found no co-localization between the modified vectors and the Golgi (Figure 2B), suggesting that these engineered vectors were not blocked in the Golgi. To clarify whether the RGD-modified virus was trapped in the endosome, HeLa cells overexpressing either red fluorescent protein-conjugated RAB5 or RAB7, early and late endosome markers (36), were transduced with Y77-RGD and Y116-RGD vectors. Six hours later, viral vectors inside cells were examined by immunocytochemistry and confocal microscopy. We found that viral particles co-localized with endosomes (Figure 2B and Supplementary Figure S3B), indicating their blockage in this organelle. We thus concluded that modifications of lentiviral vectors at residues Y77 or Y116 cause the vectors to remain in the endosome.

With the aim to determine whether the viral integration occurred within 120 h after transduction in 293T cells with wild-type (WT), Y77-RGD, Y116-RGD or D192-RGD modified vectors, the genomic DNAs of the transduced 293T cells were harvested as representative templates for PCR amplification. We found that no PCR product was detected for Y77-RGD and Y116-RGD vector (Figure 2C). This supports the interpretation that conjugation of RGD ligand at both Y77 and Y116 residues forced lentiviral vectors to remain in the cytoplasm, preventing viral integration into the host genome. In addition, these results were further supported by qualitative data indicating the viral transduction capability (Supplementary Figure S4A). The effects of RGD modifications on viral transduction efficacy were further quantitatively analyzed by measuring the relative luciferase activities. This transduction indicator for the Y77-RGD and Y116-RGD groups showed <1 and 3%, respectively, of the value of the unmodified Y77 and Y116 vectors at 9 days after transduction. By contrast, at day 9, there was no substantial difference in the transduction indicator for either WT vectors or D192 after RGD-modification from their levels at day 2 (Figure 2D; Supplementary Figure S4B and C). Thus, modification of the Y77/Y116 residues can be clearly seen to directly result in trapping the virus inside the endosome, thus preventing their integration into the genome and resulting in the loss of long-term expression capability.

### Design and synthesis of a photo-cleavable molecules for constructing a photo-switchable lentiviral vector

**Figure 3.**
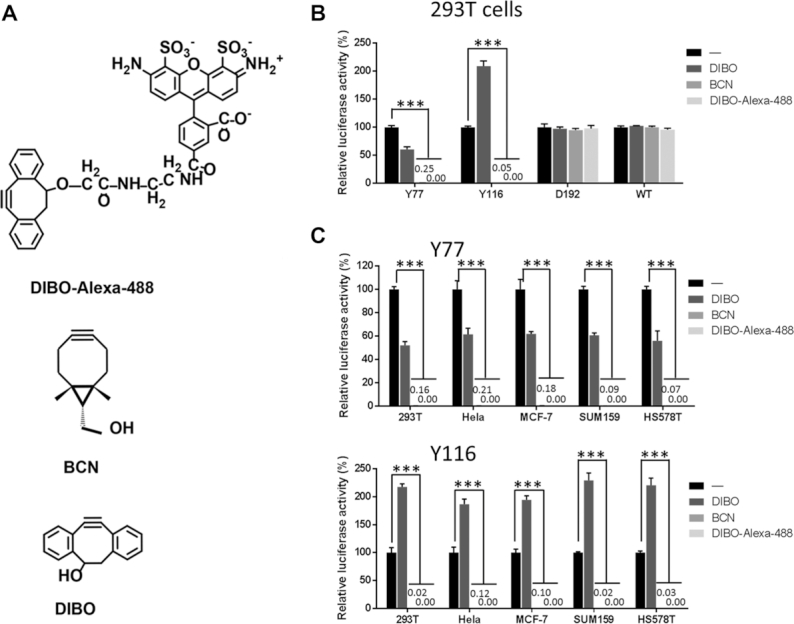
Effects of DIBO, BCN and DIBO-Alexa Fluor-488 conjugation on viral transduction capability. (**A**) Chemical structures of DIBO, BCN and DIBO-Alexa Fluor-488. (**B**) Effects of DIBO, BCN and DIBO-Alexa Fluor-488 conjugation on lentiviral vector transduction capability in 293T cells. (**C**), Effects of DIBO, BCN and DIBO-Alexa Fluor-488 conjugations at residue Y77 or Y116 on lentiviral vector transduction capability in five different cell lines. Viral transduction capability was measured by relative luciferase activity. The expression levels of BCN and DIBO-Alexa 488 were too small to see at this scale. ‘-’ indicates no conjugation as negative control. ***P < 0.01 versus corresponding control, *n* = 3.

**Figure 4.**
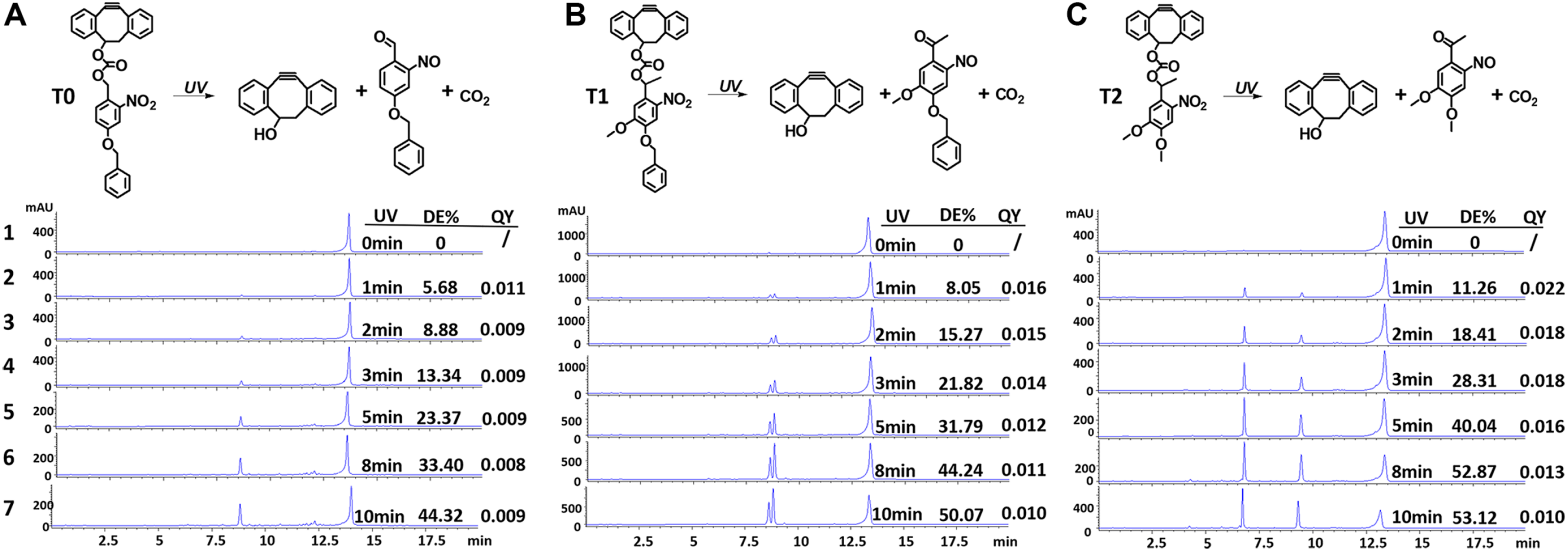
Design and identification of photo-cleavable molecules. (**A**–**C**), Photo-induced cleavage of T0, T1 and T2. Analysis of decaging efficiency of photo-cleavable molecules by HPLC. T0, T1 and T2 were irradiated with UV light (365 nm, 7 mW/cm^2^) for different periods of exposure. Line 1: no irradiation; 2–7, T0, T1 and T2 were irradiated with UV light for 1, 2, 3, 5, 8 and 10 min, respectively. DE%, decaging efficiency. QY, quantum yield.

Inspired by the above findings, we set out to design a molecule conjugated to the Y77 and Y116 residues through a photo-cleavable linkage to initially block transduction of the lentiviral vector. After exposure of the chimeric viral vector to UVA light (365 nm) the photo-caging group could be subsequently removed, thus restoring full viral transduction capability. We hypothesized that transduction of this photo-switchable viral vector can be precisely manipulated by ultraviolet radiation in a temporally and spatially controlled manner for targeted gene therapy. To accomplish synthesis of the photo-switchable blocking group described above, the light-cleavable molecule must contain an alkyne functionality for the site-specific conjugation to an azido-modified viral protein via click chemistry. It must also contain a photo-cleavable linkage group that can be effectively severed by UV irradiation, and an additional group that completely blocks viral transduction capacity before photocleavage. In actuality, the covalent bond formed between alkyne and azido groups likely remains on the viral surface after UV irradiation, presenting a situation that may affect the outcome of the transduction pathway. DIBO and BCN are two alkyne groups commonly used in copper-free click chemistry reactions (Figure 3A). We first examined whether their presence affected the viral transduction capability of Y77 or Y116 vectors, using wild-type and D192 vectors as controls. We found that modification with DIBO or BCN had no effect on the transduction capability of the control viruses. However, conjugation of DIBO at Y77 decreased transduction efficiency by approximately 40%, whereas the BCN modification almost completely blocked transduction. In contrast, DIBO modification at Y116 resulted in a 2-fold increase in viral transduction capability, whereas the BCN modification blocked viral transduction (Figure 3B). These results were confirmed in HeLa, MCF-7, SUM159 and HS578T cells (Figure 3C). We, therefore, selected DIBO as the alkyne group portion of the photo-cleavable molecule. To meet the requirements of the photo-switchable lentiviral vector, we first synthesized the photo-cleavable molecule T0 containing the DIBO functionality and a photosensitive group comprising an *o*-nitrobenzyl-type parent nucleus, and a benzyl group, that ensures that the photo-cleavable molecule is of a certain size (detailed information on synthesis can be found in the Supplementary Data). The decaging efficiency of T0 exposed to UV light for up to 10 min was only 44% (Figure 4A). We, therefore, synthesized another photo-cleavable molecule T1, containing a more efficient photo-cleavable group and found that it exhibited a higher photo-cleavage efficiency, attaining up to 50% after 10 min of UV irradiation (Figure 4B). Although this was lower than the previously reported value (37), we speculated that the aromatic structure affects UV absorption and slows down light-induced breakage. We, therefore, removed the aromatic ring constituent and selected the photosensitive o-nitrobenzyl moiety with two methoxy groups instead and synthesized the photo-cleavable molecule T2 (Figure 4C). The decaging efficiency of T2 exposed to UV light for up to 10 min was 53%, which was slightly higher than the decaging efficiency of T1 (50%). Moreover, the quantum yield that means decaging events per absorption event was characterized ranging from 0.008 to 0.022 (Figure 4A–C).

**Figure 5.**
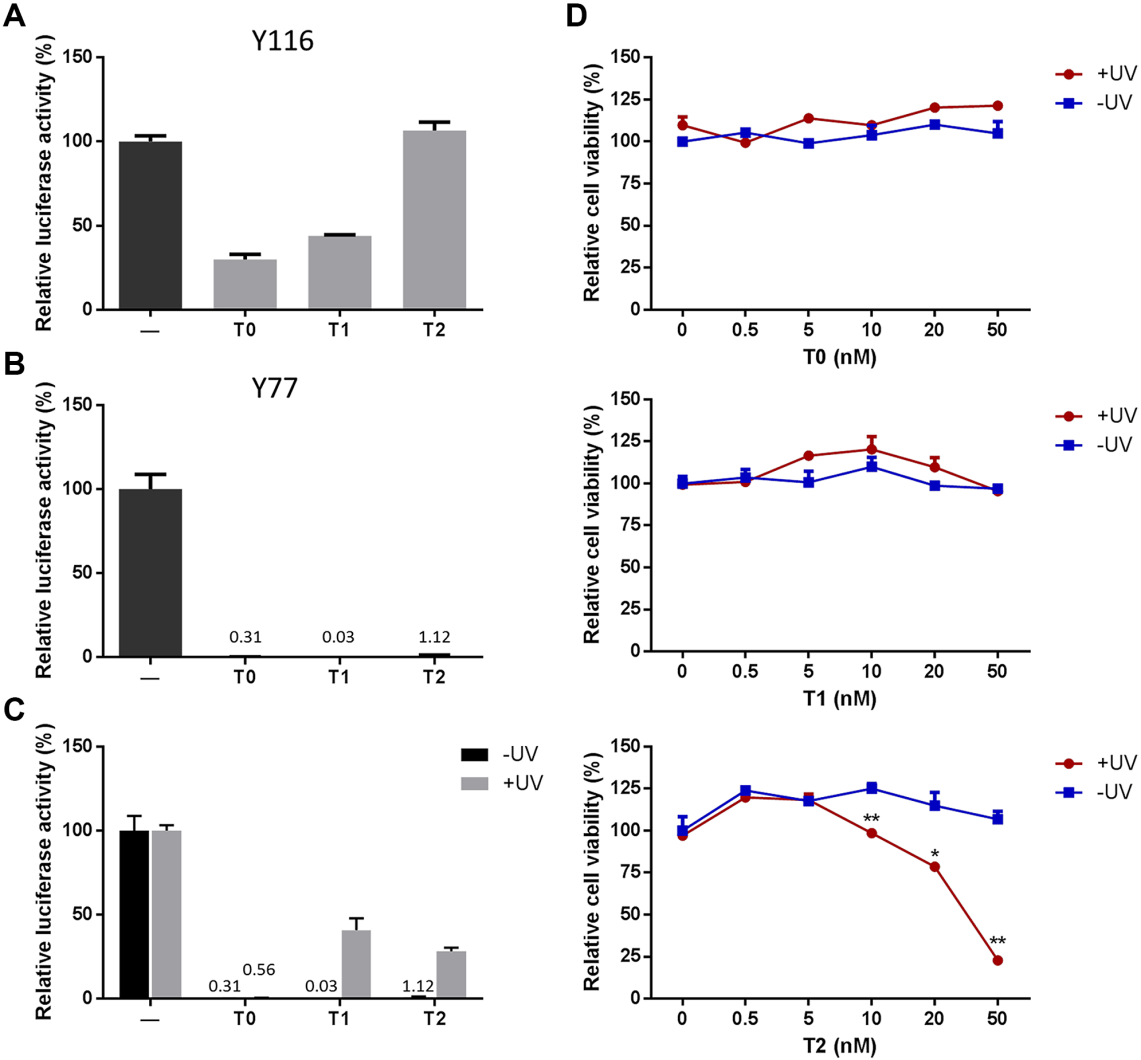
Evaluation of function and toxicity of photo-cleavable molecules T0, T1 and T2. (**A**) Transduction capability of lentiviral vectors with T0, T1 and T2 conjugated at residue Y116. The lentiviral vector was packaged with the luciferase reporter gene. Viral transduction capability was measured by relative luciferase activity. ‘-’ indicates no conjugation as negative control. (**B**) Transduction ability of lentiviral vectors with T0, T1 and T2 conjugated at residue Y77. (**C**) Photo-switchable gene delivery by lentiviral vector conjugated with T0, T1 and T2 at residue Y77 (Y77-T0, Y77-T1 and Y77-T2, respectively). (**D**) Cytotoxicity of T0, T1 and T2, at various concentrations of the photo-cleavable linker, with or without UV irradiation. **P < 0.01, *P < 0.05 versus corresponding control, *n* = 3.

To verify the blocking effect of light-cleavable molecules on viral transduction, T0, T1 and T2 were conjugated to viral vectors Y77 and Y116. All three photolinker molecules blocked the transduction of Y77 but not the transduction of Y116 virus (Figure 5A and B). We, therefore, used the former for subsequent photo-switch experiments. The transduction capability of Y77-T0 virus was not restored by UV irradiation due to its low bond splitting efficiency. In contrast, UV light exposure restored the transduction capability of the Y77-T1 and Y77-T2 viruses to 40.8 and 28.4% of the value of the Y77-DIBO virus, respectively. Viruses that were not exposed to UV radiation had no transduction activity (Figure 5C). These results demonstrate that the transduction capability of Y77 virus modified by T1 and T2 could be restored by light irradiation. In the above experiment, we observed that many cells died in the Y77-T2 UV irradiation group, suggesting that a toxic product was generated by T2 fragmentation. We, therefore, evaluated the cytotoxicity of the three compounds (from 0 to 50 nM) and found that T0 and T1 were not cytotoxic at the tested concentrations irrespective of light exposure. However, although T2 was not cytotoxic in itself, increasing cytotoxicity was observed as it decomposed following UV irradiation. At 50 nM T2 concentration, cell viability decreased to about 20% of the value in the control group (Figure 5D). Thus, taking into consideration the blocking effect and safety, T1 showed the best performance of the three light-cleavable molecules. In conclusion, the Y77-T1 vector was found to exhibit the best combination of characteristics, among all these modified lentiviral vectors we tested, that are suitable for use as a photo-switchable lentiviral vector.

Given that RGD conjugation blocks viral transduction because of its being retained in the endosome for a long period, we investigated whether the T1 substitution also has the same effect. Fluorescent images and luciferase activity analyses of the T1 modified lentiviral vectors are shown in Supplementary Figure S5A, C, D and E. These data clearly show that T1 modification at the Y77 residues also results in blocking long-term viral transduction. Furthermore, PCR amplification of GFP sequences extracted from genomic DNA of 293T cells at 120 h post transduction confirmed that T1 blocked integration of modified Y77 virus. However, T1 on D192 and Y116 viruses did not block integration (Supplementary Figure S5B). Moreover, co-localization analysis at 6 h after transduction showed that Y77-T1 virus was indeed blocked in the endosome and not in the Golgi (Supplementary Figure S6), indicating that the blocking mechanism of Y77-T1viral vectors is similar to that of Y77-RGD. Thus, conjugation of T1 appears to have the same effect in blocking viral transduction as conjugation of RGD.

### Temporal and spatial control of transduction *in vitro* using the photo-switchable lentiviral vector

**Figure 6.**
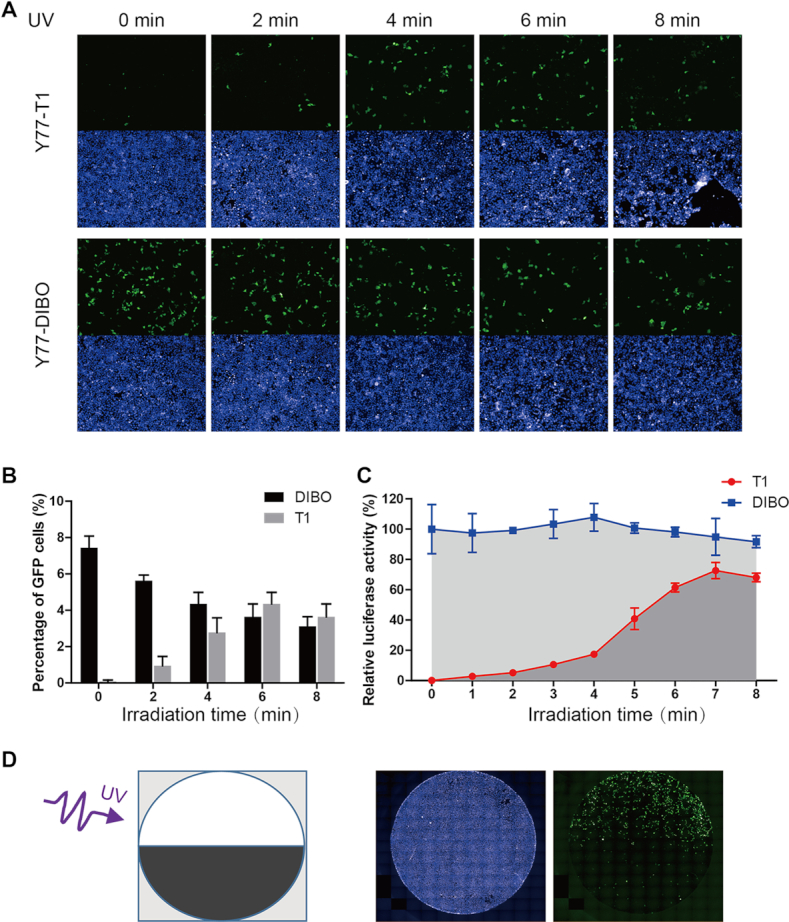
Characteristics of temporal and spatial control over transduction capability of photo-switchable lentiviral vectors in 293T cells. (**A**) Characterization of the effect of different irradiation times on the recovery of viral transduction capability based on GFP reporter expression. Green fluorescence identifies virus-transduced cells; nuclei stained with DAPI appear blue. Lentivirus modified by DIBO served as a control. (**B**) Quantitative analysis of the effects of irradiation time on GFP delivery by Y77-T1 and Y77-DIBO vectors by flow cytometry. (**C**) Characterization of the effect of different irradiation times on recovery of virus transduction capability measured by the relative luminescence due to the packaged luciferase reporter gene. Transduction ability was measured as relative luciferase activity. (**D**) Photo-induced spatially specific transduction of cells by the Y77-T1 viral vector. A schematic illustration of the spatial irradiation protocol (left) and the results (right) are shown.

We next investigated whether the Y77-T1 transduction capability can gradually be restored after increasing irradiance in a host cell system (293 T cells). The Y77-T1 virus containing the *GFP* reporter gene had no transduction capability in the absence of UV irradiation, but its transduction capability gradually recovered in a time-dependent manner upon UV exposure to in vitro cell cultures (Figure 6A and B). A similar result was obtained using a luciferase gene-containing Y77-T1 virus. After 7 min of UV exposure, viral transduction capacity reached a maximum that was ∼73% of the value for the Y77-DIBO virus. However, the transduction ability of both Y77-T1 and Y77-DIBO viruses declined after 8 min of irradiation, possibly due to long-term light damage to the cells and viruses (Figure 6C). These results suggested that the outcome of viral transduction of Y77-T1 can be manipulated in a time-dependent manner.

To monitor the spatial specificity of Y77-T1, we observed viral transduction with a fluorescence microscope. Green fluorescence was visible in the top half of the plate that had been irradiated with UV light for 5 min, whereas no signal was detected in the bottom half of the plate absent the UV radiation (Figure 6D). These results indicate that the Y77-T1 virus can be used to achieve locus specific, target cell transduction of GFP by exerting spatial control over photo-induction.

Light at a wavelength of 365 nm has been used in biological studies and the treatment of vitiligo (38,39). Nevertheless, we found obvious toxicity during extended time exposure to this wavelength. To assess the relationship between toxicity and irradiation intensity both in human cells and viruses, 293T cells were transduced with WT, Y77 and Y116 viral vectors (loaded with luciferase) then irradiated for different time intervals. Luciferase activity in the cells was measured after 72 h. The transduction capability of all three viruses decreased with irradiation time (Supplementary Figure S7A). We also found that irradiation times shorter than 6 min with 7 mW/cm^2^ had no effect on cell viability. A significant decrease was observed after 7 min of irradiation (Supplementary Figure S7B). Therefore, irradiation for 6 min was found to be safe and can effectively activate the photo-switchable viral vector.

### Temporal and spatial properties of the photo-switchable lentiviral vector *in vivo*

**Figure 7.**
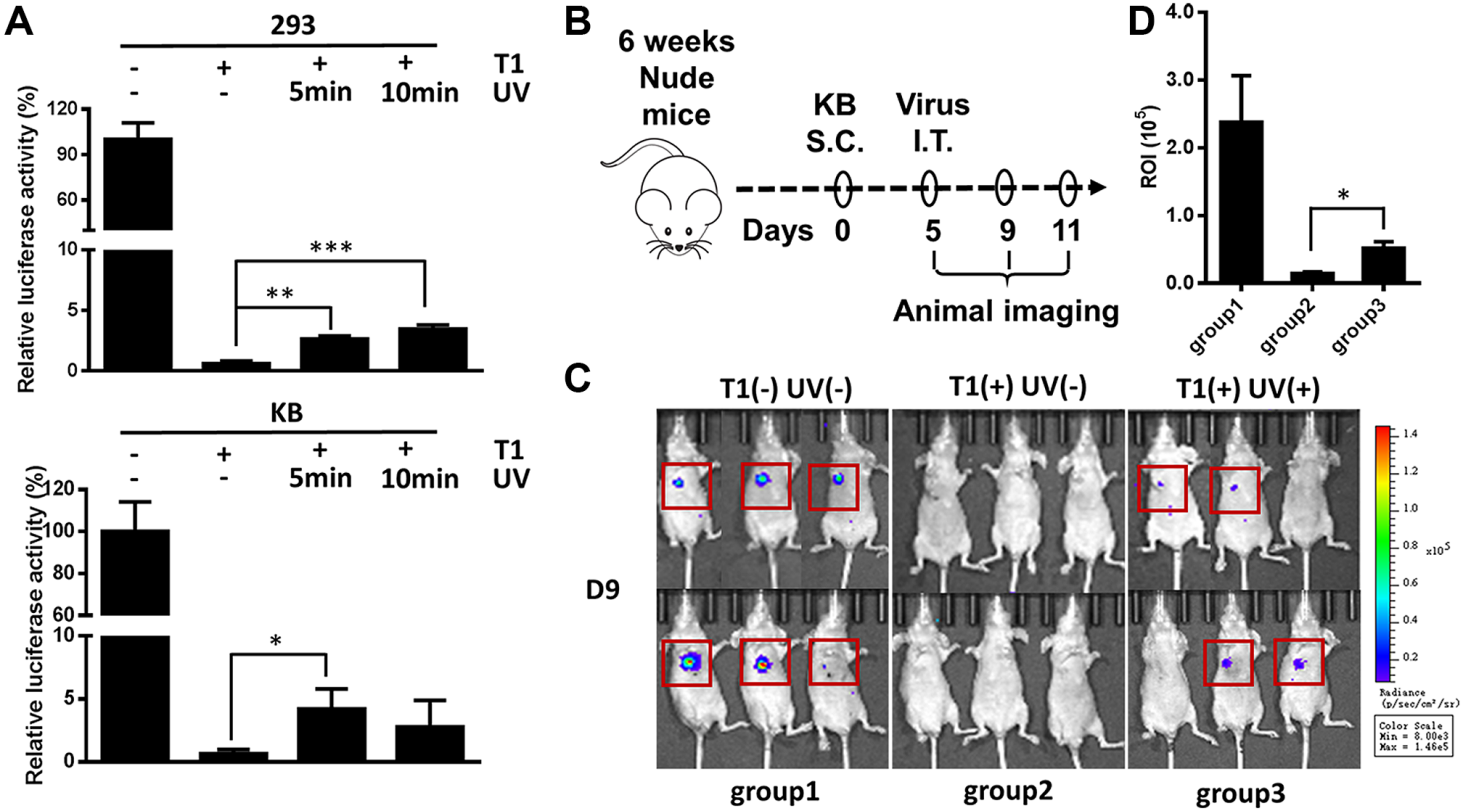
Characteristics of temporal and spatial control over transduction capability of photo-switchable lentiviral vectors *in vivo*. (**A**) The recovery of Y77-T1 virus transduction capability induced by transmitted UV through mouse skin *in vitro*. Viral transduction capability was measured by relative luciferase reporter expression in 293T cells and KB cells (*P < 0.05, **P < 0.01, ***P < 0.001, *n* = 3). (**B**) Flowchart of the *in vivo* subcutaneous transplantation tumor model for evaluation of transduction capacity of the photo-switchable lentiviral vector. S.C: subcutaneous injection; I.T: intratumoral injections. (**C**) Bioluminescence imaging of nude mice inoculated with KB tumor cells at the right flank. T1(−) UV(−): group1, Y77 virus and no UV irradiation. T1(+) UV(−): group2, Y77-T1 virus and no UV irradiation; T1(+) UV(+): group3, Y77-T1 virus and 5 min of UV irradiation. There were six mice per group. (**D**) ROI analysis of the bioluminescence imaging in (C) (*P< 0.05, *n* = 6).

To further explore the temporal and spatial properties of the photo-switchable lentiviral vector *in vivo*, we sought to determine whether UVA light is able to penetrate the mouse skin to activate the Y77-T1 vector. To investigate this, 293T and KB cells were transduced with Y77-T1 and irradiated with UV light for 5 or 10 min through the skin of nude mice. Viral transduction capacity was measured after 72 h by the luciferase assay. The luminescence signals of 293T cells irradiated with UV for 5 min and 10 was 4.46- and 5.87-fold that of the non-irradiated group, respectively. Similarly, in KB cells, the luminescence signal of the group irradiated with UV for 5 min was 6.67-fold that of the non-irradiated group. However, exposure of KB cells to UV radiation at 365 nm for 10 min led to no difference in measured luminescence as compared with the non-irradiated groups, due to cellular toxicity upon longer UV exposure that leads to fewer surviving cells (Figure 7A). These data suggested that a fraction of 365 nm light is still capable of penetrating the skin of mice, despite its being less than a few millimeters thick, and thus able to induce photo-uncaging of the Y77-T1 vector to complete the transduction process.

As a proof-of-concept of the photo-switchable viral vector *in vivo*, we further focused on this animal model since UV light was shown to partially penetrate the skin of nude mice. As shown in Figure 7A, KB cells showed relatively greater effective transduction yields for Y77-T1 vectors than did 293T cells, and KB cells could be more easily transplanted in the mice via subcutaneous injection as well. Thus, KB cells were used in all remaining experiments to establish the subcutaneous transplantation tumor model. The *in vivo* test of the photo-switchable lentiviral vector was carried out as described in the ‘Materials and Methods’ section (and summarized in Figure 7B). Bioluminescence images of nude mice were taken at the 9th day after subcutaneous inoculation of KB cells. All six mice in group 1 injected with Y77 virus without UV irradiation (positive control) showed a strong luminescence signal, while zero of the six mice in group 2 injected with Y77-T1 virus that was left uncaged, having received no UV radiation (negative control), showed a luminescence signal. Among the mice in group 3 that were injected with Y77-T1 virus and received 5 min of UV irradiation, four out of six showed a positive luminescence signal and two showed no luminescence, which may have been due to the low efficiency of UV penetration through the skin. Consistent with the results obtained *in vitro*, bioluminescence imaging of nude mice showed that the Y77-T1 virus vector presented no transduction capability in the absence of UV irradiation, and that its transduction capability was restored after 5 min of UV exposure (Figure 7C). Region of interest (ROI) analysis of the bioluminescence images in Figure 7C showed that the luminescence signal of the group irradiated with UV was 3.56-fold compared to that of the non-irradiated group (Figure 7D). These results indicate that the transduction of Y77-T1 can be spatially and temporally controlled *in vivo* by exposure to UV radiation.

## DISCUSSION

The lack of control over processing of viral vectors by cells during transduction events post engulfment and phagocytosis presents a significant obstacle to realizing their broad applications for gene delivery. With an aim to address this issue in this proof-of-concept study, we designed a controllable lentiviral vector with a molecular structure allowing for temporary and reversible inactivation of its transduction capability. This vector modification is based on the vital platform we previously developed for the site-specific incorporation of unnatural amino acids, specifically NAEK, into a coat protein of lentiviral vectors. The necessary structural modification requires conjugation to a photochemical ligand via a NAEK-mediated click chemistry reaction (35). In this study, we first identified the most appropriate sites on VSVg envelope protein to conjugate to a steric blockage group through a linker moiety (specifically a photolabile ortho-nitrobenzyl linker) that has the capability of temporarily blocking viral transduction. We found that modifications at residues Y77 and Y116, located within the membrane fusion domain, are effective at holding up lentiviral transduction (Figure 1), probably blocking the fusion of the virus membrane to host cell endosomes. The same modification at the Y73 locus, despite being located within the same membrane fusion domain, was not effective as the other blocking site. This is due to the poor incorporation of NAEK at this site within a truncated length of VSVg protein according to our previous study (35) and thus no Y73-NAEK-modified viral vector could be produced. Additionally, we found that a chimeric vector was generated from modification of residue D121, which is also within the fusion domain. However, the full transduction capability was not blocked by introduction of the photolabile moiety (Figure 1). These results indicate that there are some loci and types of modifications within the fusion domain of VSVg, but not all modifications within this domain, that affect the fusion function.

Generation of the photo-decaged lentiviral vector relies on a click chemistry reaction between the azide group of the site-localized NAEK molecule and an alkyne group inside the light-cleavable cage molecule, forming a triazole covalent bond linking NAEK to the photolabile cage. Upon exposure to UV light, the photolabile cage on the viral vector is removed, but the triazole bond to the VSVg protein residue remains. In this study, DIBO and BCN were utilized as the alkyne-carrying moieties. Due to the smaller size of BCN, it was initially believed that introduction of BCN-carrying cage would have less effect on viral transduction. However, modifications by BCN at Y77 and Y116 both completely blocked transduction of the lentiviral vector, whereas vector variants derived from DIBO at Y77 just blocked viral transduction to a lesser degree, as shown in Figure 3. An additional unexpected observation is that introduction of the DIBO-carrying cage at Y116, rather than blocking viral transduction, actually increased viral transduction approximately 2-fold. We speculate that this reflects the possibility that aromatic DIBO-mediated alteration of the vector's hydrophobicity that affects the capacity for membrane fusion and is consistent with previous reports (33,34,40,41). We therefore conclude that the chemical structure and its (hydrogen and π) bond-forming configurations, rather than the molecular size of the cage plays the important role in influencing viral transduction. These might explain the mechanism underlying the restricted photocleavage efficiency of the light-cleavable cages T0, T1 or T2 to release lentiviral transduction from endosomes and produce gene product in high yields. Of course, steric hindrance also plays a significant role as larger cages such as DIBO-RGD, -FA and -Alexa Fluor-488 can completely block vector transduction (Figure 1D and E; Supplementary Figure S1F). Thus, there is considerable freedom to design cages to modify the pseudotyped VSVg according to different intended purposes.

The method described here provides a direction for potential translational applications of lentiviral vectors, which despite showing promise has some limitations. One major concern is the wavelength of light, ∼365 nm used in this study to uncage the lentiviral vector. The ortho-nitrobenzyl ester selected as the photolabile group is suitable for photoirradiation at 365 nm and exerts efficient and rapid cleavage. For a proof-of-concept study, our results do indicate that the use of the photo-inducible bond cleavage can reverse lentiviral vectors and thus enable regulation of lentiviral vectors in a switchable manner. However, such a wavelength of light is indeed not clinically ideal due to its poor penetration into tissue (less than a few millimeters) and also because of its ability to induce DNA mutations (42). Despite the fact that a large series of photolabile protecting groups have been explored over the last decade, a paramount long-term goal still remains: i.e. to find a red-shifted, photo-release agent that is activated within the therapeutic window, ∼650–950 nm, enabling deeper tissue penetration (43,44), while eliminating exposure to mutation associated light sources. Optimization of the photo labile o-nitrobenzyl derivatives to adapt to red-shifted illumination sources for sensitive, two-photon photo-cleavage clearly has a long way to go to achieve desirable efficiencies (45,46). An alternative approach that addresses the penetration issue is to utilize fiber-optic probes or UV sources, such as implanted LEDs (47) that uncage vectors under the guidance of light pulses in parts of the body only where they’re needed. Photo-switchable options by such approaches have improved over the years but they are all still inadequate.

In conclusion, we report here an approach for generation of a caged lentiviral vector via insertion of an unnatural amino acid at specific loci of a pseudotyped VSV coat protein for photo-switchable transduction of a target gene. Expansion of the genetic code of a lentiviral vector makes it possible to incorporate an azido-containing NAEK site-specifically on its envelope and coupled to a cage structure through a photo-labile linker by means of click chemistry. The cage prevents integration of the vector by trapping it inside the endosome until exposure to UVA light which subsequently removes the photo-caging group and thus restores the transduction capability of the lentiviral vector. Our results show that severance of the photo-labile bond can release lentiviral vectors and thus regulate the viral transduction (integration) in a switchable manner. The caging-uncaging strategy proposed herein allows targeted delivery to locations where the stimulus is present while simultaneously reducing off-target delivery to areas where the stimulus is absent, thereby enabling a safer treatment and requiring lower administration dosages. Our photo-switchable lentiviral vector can be used to control the level and location of transgene expression in a population of cells *ex vivo*, promoting the development of artificial organs and tissue engineering for regenerative medicine applications. Controllable gene delivery induced by light exposure is also useful *in vivo* when the disease location is known and close to the skin surface. Moreover, the benefits of temporal control over photocleavage may assist in synchronizing gene integration among a population of host cells, making the photo-switchable lentiviral vector a useful tool for mechanistic studies of transduction in cells. The methods presented here may become a useful and general approach for generating a set of photo- and other forms of switchable vectors that can be adapted to other viral carriers.

## Supplementary Material

gkz659_Supplemental_File
