# Supplementary material for: Generation of a caged lentiviral vector through an unnatural amino acid for photo-switchable transduction
Source: Nucleic Acids Res. 2019 Jul 30;47(19):e114. doi: 10.1093/nar/gkz659 (PMC6821241; doi:10.1093/nar/gkz659)
Supplement: gkz659_Supplemental_File [file gkz659_supplemental_file.pdf]

SUPPORTING INFORMATION

Generation of a Caged Lentiviral Vector Through an Unnatural Amino Acid for Photo-Switchable Transduction

Yan Wang[1, 2#], Shuai Li[1, 2#], Zhenyu Tian[1, 2], Jiaqi Sun[1, 2], Shuobin Liang[1, 2], Bo Zhang[4], Lu Bai[1, 2], Yuanjie Zhang[1, 2], Xueying Zhou[1, 2], Sulong Xiao[1, 2], Qiang Zhang[1, 3], Lihe Zhang[1], Chuanling Zhang[1, 2]*, Demin Zhou[1,2]*

[1]State Key Laboratory of Natural and Biomimetic Drugs, School of Pharmaceutical Sciences, Peking University, Beijing 100191, China

[2]Department of Molecular and Cellular Pharmacology, School of Pharmaceutical Sciences, Peking University, Beijing 100191, China

[3]Department of Pharmaceutics, School of Pharmaceutical Sciences, Peking University, Beijing 100191, China

[4]Center for Translational Medicine, Chinese Academy of Medical Sciences and Peking Union Medical College, Beijing 100730, China

[#]These authors contributed equally this work.

*Address correspondence to:
Prof. Demin Zhou and Dr. Chuanling Zhang. School of Pharmaceutical Sciences, Peking University, No. 38, Xueyuan Road, Beijing 100191, China. Tel: 86-10-8280-5857; fax: 86-10-8280-5519; email: deminzhou@bjmu.edu.cn, zhangchuanling@bjmu.edu.cn

Supplementary Figures:

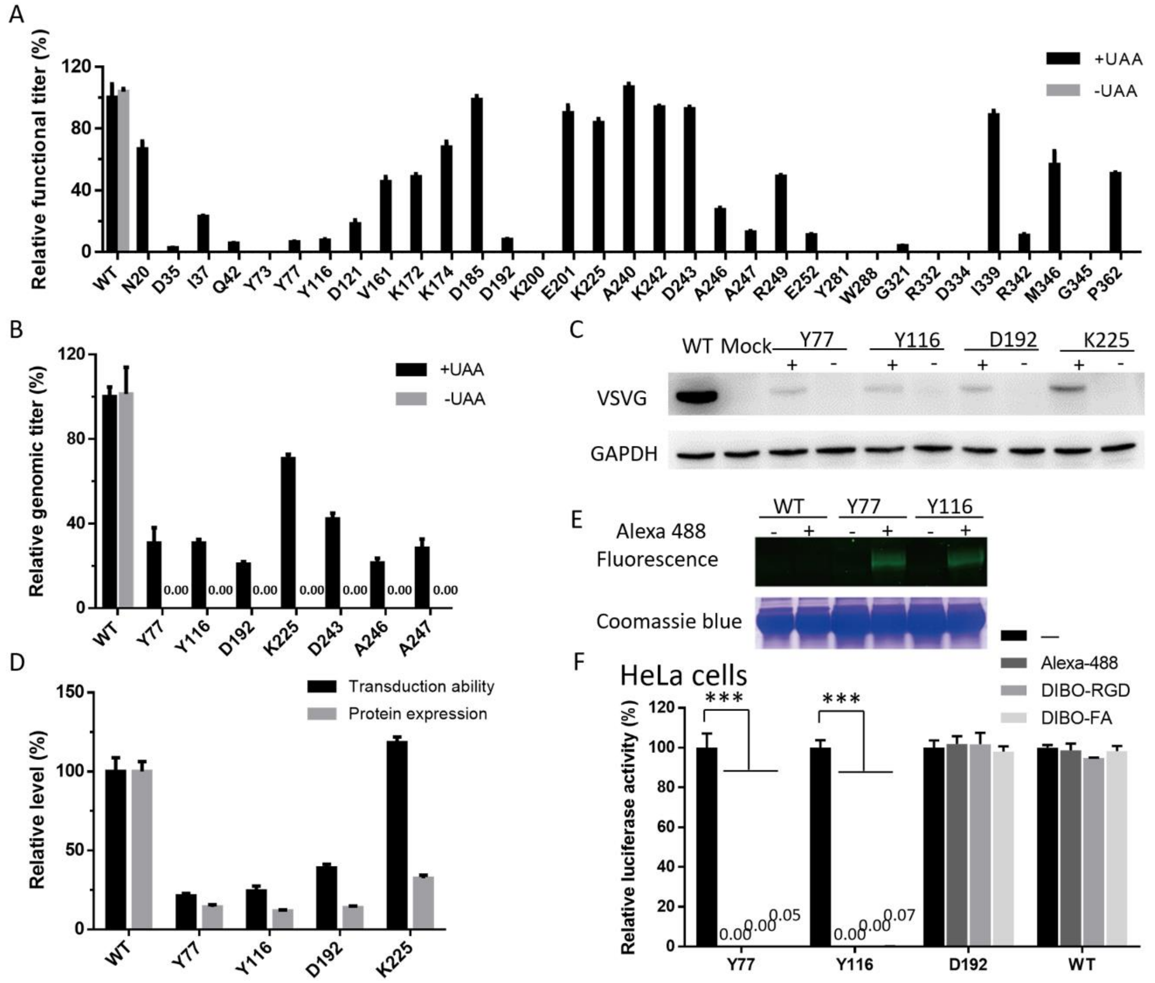

Supplementary Figure 1. Site identification and conjugation of NAEK to lentiviral vector. **A**, Relative functional titers of different mutant lentiviral vectors produced with or without unnatural amino acid (UAA). Viral functional titers were measured by the luciferase assay and normalized to that of the wild-type lentiviral vector. **B,** Relative genomic titers of different mutant lentiviral vectors characterized by real-time RT-PCR. **C**, The exploitation of genetic code expansion-mediated unnatural amino acid incorporation into lentiviral envelope protein at different sites. The expression level of VSVg or GAPDH was analyzed by western blotting. D, Comparisons of the effect of UAA incorporation at different sites on expression of VSVg protein and viral transduction ability of progeny vectors. The viral transduction ability was measured by the ratios of the functional titers to the genomic titers and normalized to that of the wild-type lentiviral vector. The relative protein expression is from the quantitative analysis of the figure S1C. **E**, Click chemical conjugation of Y77- and Y116-Alexa Fluor-488 confirmed by SDS-PAGE, fluorescence imaging and Coomassie Brilliant Blue staining. **F**, Transduction ability of the Alexa Fluor-488, cRGD, or

FA-modified lentiviral vector in HeLa cells. All quantitative data shown are average values with standard deviations from triplicate experiments (***$P<0.001$). "-" indicates no conjugation as a control.

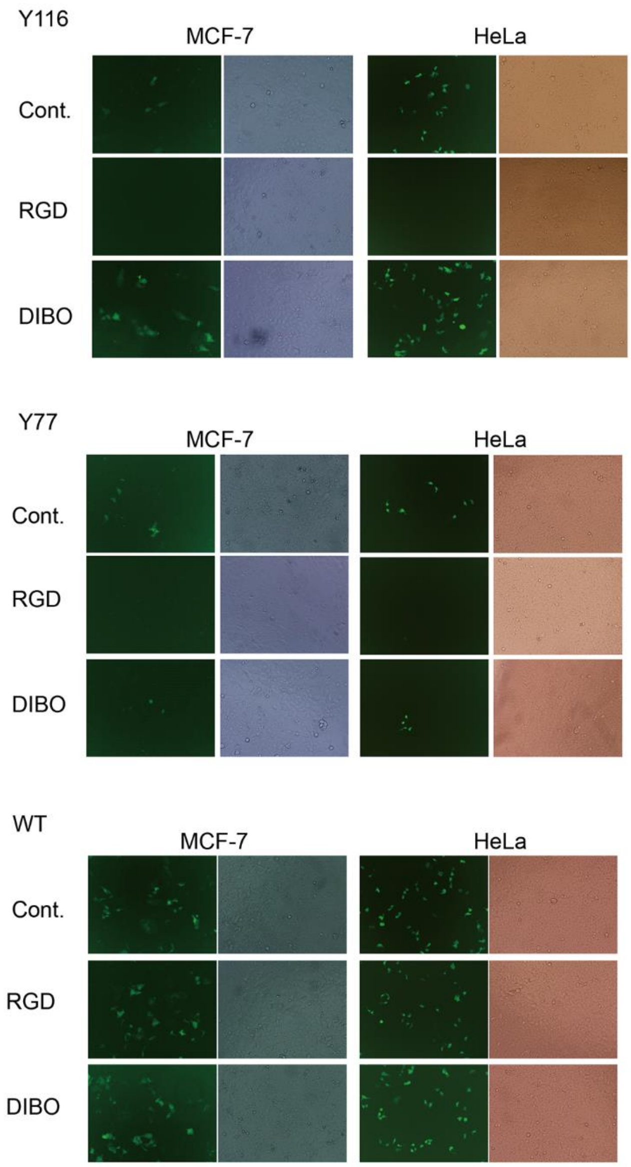

Supplementary Figure 2. Fluorescence micrographs of cells transduced with RGD-modified lentiviral vectors harboring the GFP reporter gene. HeLa and MCF-7 cells were transduced with wild-type lentiviral vector or vector conjugated with RGD, DIBO, at residues Y77 and Y116, and fluorescence images were acquired with a fluorescence microscope (Nikon, Tokyo, Japan) 48 h after transduction. Green fluorescence identifies virus-transduced cells. Cont. indicates no conjugations as a control.

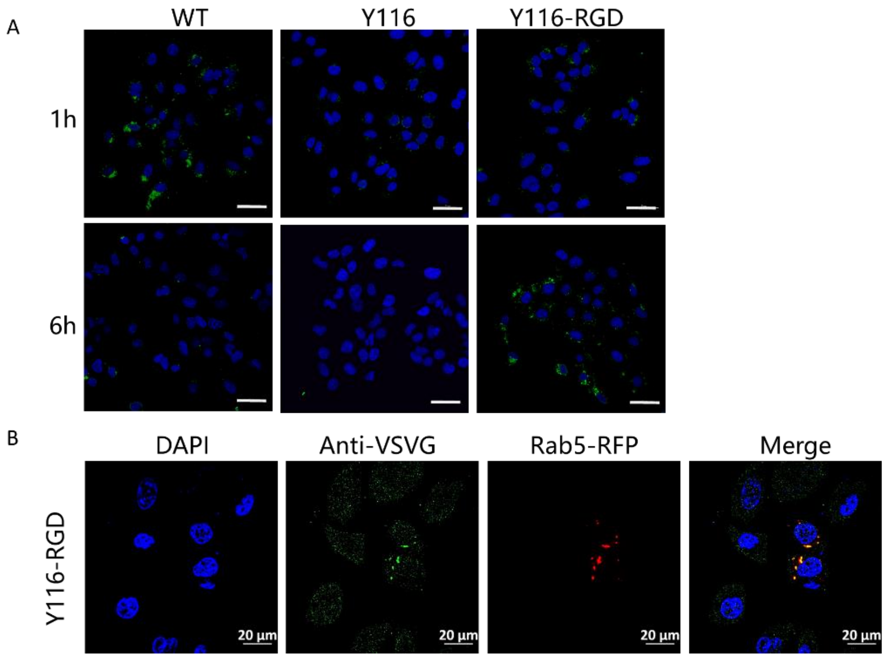

Supplementary Figure 3. Transduction pathway analysis of lentiviral vector modified with RGD at residue Y116 (Y116-RGD). A. Subcellular localization analysis of Y116-RGD 1 and 6 h after viral transduction in HeLa cells. Scale bar, 50 μm. **B.** Co-localization analysis of Y116-RGD (green) and Rab5-red fluorescent protein (RFP) (red) marking early endosomes in HeLa cells 6 h after viral transduction. Scale bar, 20 μm.

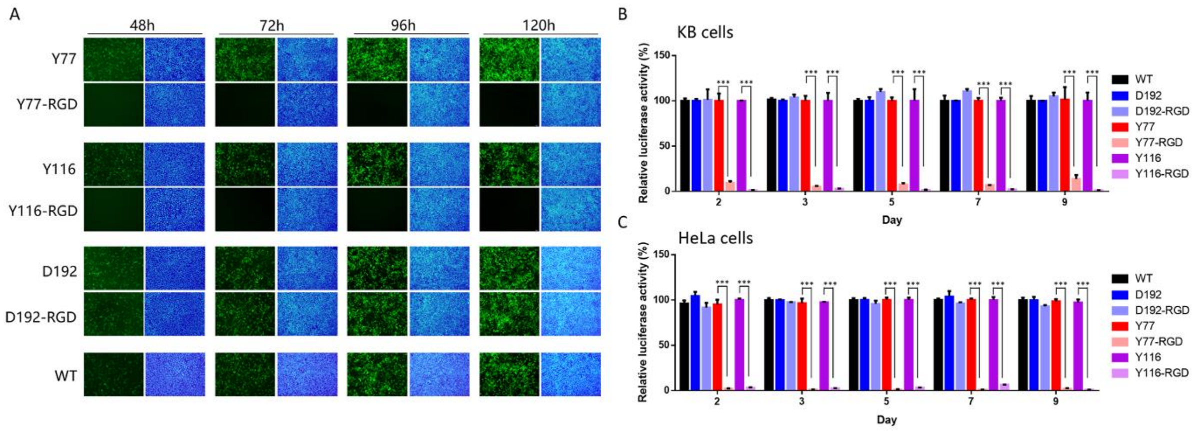

Supplementary Figure 4. Transduction activity of RGD-modified lentiviral vectors.

A. Qualitative analysis of the effects of RGD modifications on viral transduction ability. 293T cells were transduced with wild-type lentiviral vector or a vector conjugated with RGD at residues Y77, Y116, and D192, and fluorescence images were acquired with a fluorescence microscope (Nikon, Tokyo, Japan) 48, 72, 96, and 120 h after transduction. Green fluorescence identifies virus-transduced cells; nuclei stained with DAPI appear blue. **B**, **C,** Quantitative analysis of the effects of cRGD modifications on viral transduction ability in KB cells (B) and HeLa cells (C). The luciferase activity was tested 2, 3, 5, 7, and 9 days after transduction (***P < 0.001, n = 3).

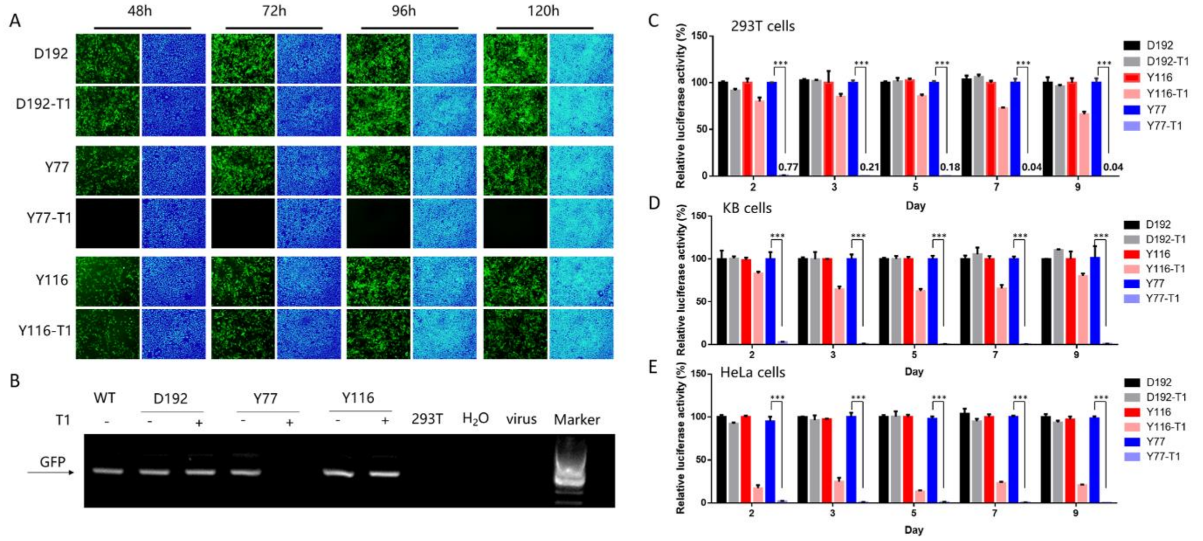

Supplementary Figure 5. Transduction and integration activity of T1-modified lentiviral vectors. A, Qualitative analysis of the effects of T1 modifications on viral transduction ability. 293T cells were transduced with a lentiviral vector conjugated with T1 at residues Y77, Y116, and D192, and fluorescent images were acquired with a fluorescence microscope (Nikon, Tokyo, Japan) 48, 72, 96, and 120 h after transduction. Green fluorescence identifies virus-transduced cells; nuclei stained with DAPI appear blue. **B**, PCR analysis of viral integration ability. PCR amplification of *GFP* sequences was performed using genomic DNA extracted from 293T cells transduced by wild-type or D192/Y77/Y116 +/- T1. Genomic DNA extracted from blank 293T and a template using water or lentivirus were used as negative controls. PCR products were evaluated on a 1% agarose gel stained with GelRed. Marker, 1-kb ladder. **C**, D, E, Quantitative analysis of the effects of T1 modifications on viral transduction ability in 293T cells (C), KB cells (D), and HeLa cells (E). The luciferase activity was tested 2, 3, 5, 7, and 9 days after transduction (***P < 0.001, n = 3).

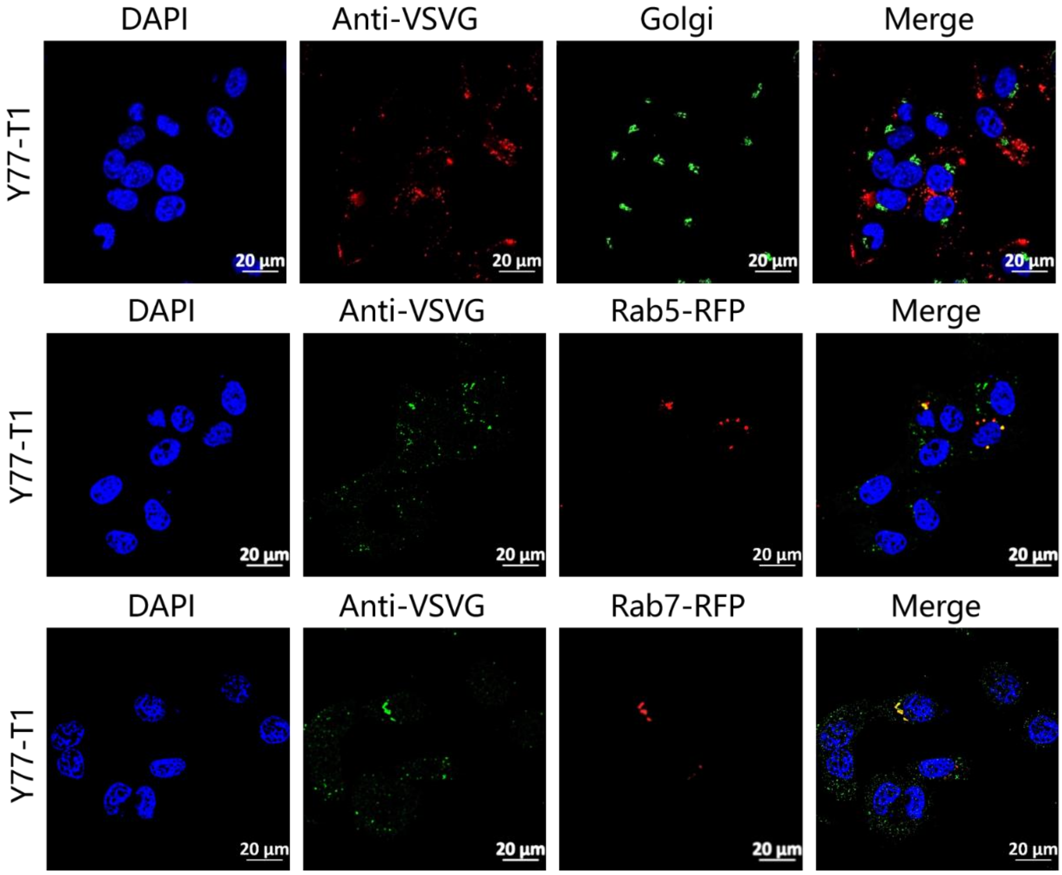

Supplementary Figure 6. Transduction pathway analysis of a lentiviral vector modified with T1 at residue Y77 (Y77-T1). Co-localization analysis of Y77-T1 (Red) and Golgi (green); Y77-T1 (green) and Rab5/7-red fluorescent protein (RFP) (red) indicate early/late endosomes in HeLa cells 6 h after viral transduction. Scale bar, 20 μm.

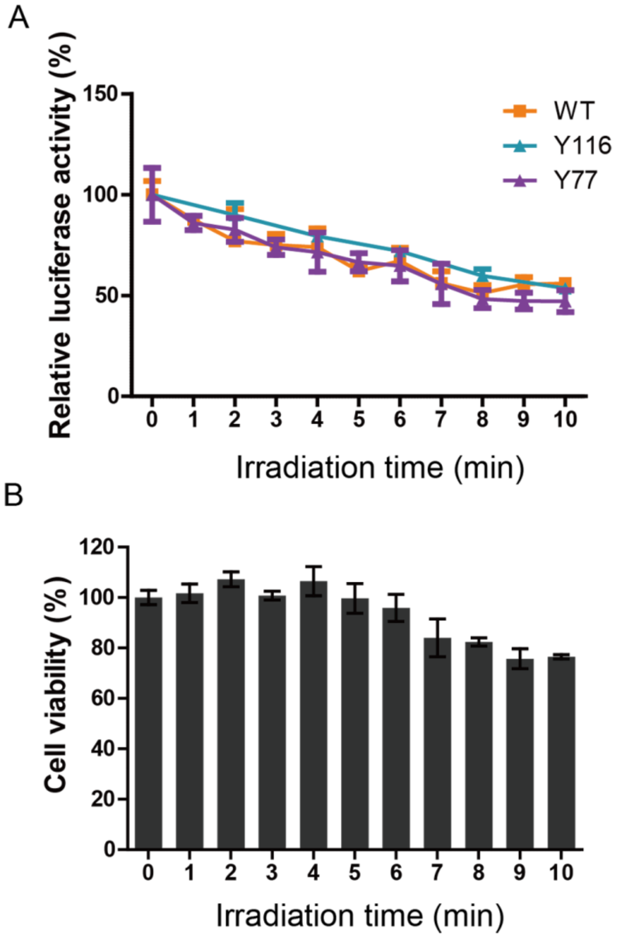

Supplementary Figure7. Toxicity analysis of UV irradiation and photo-cleavable molecules. A, Effect of ultraviolet irradiation on lentiviral transduction ability. Wild-type lentiviral vector and vectors modified with NAEK at residues Y77 and Y116 were irradiated with UV light for 0 – 10 min; 72 h after viral transduction, transduction ability was measured as relative luciferase activity. **B**, Effect of ultraviolet irradiation on cell viability. 293T cells were irradiated with UV light for 0 – 10 min; cell viability was measured 72 h after transduction.

Materials and Methods

General experimental procedures

All chemical reactions in the synthesis of the photo-cleavable linker were performed under an inert atmosphere using dry reagents and solvents. Column chromatography was performed with silica gel 60 (200–300 mesh). 1H-NMR (400 MHz) and 13C-NMR (100 MHz) spectra were taken on Bruker AVANCE III-400 spectrometers and standardized to the solvent NMR peak. Mass spectra of small molecules were obtained on a Waters Xevo TQD Mass Spectrometer using electrospray ionization (ESI). The reactions were conducted in a dark room when necessary.

The synthesis and identification of T0, T1 and T2.

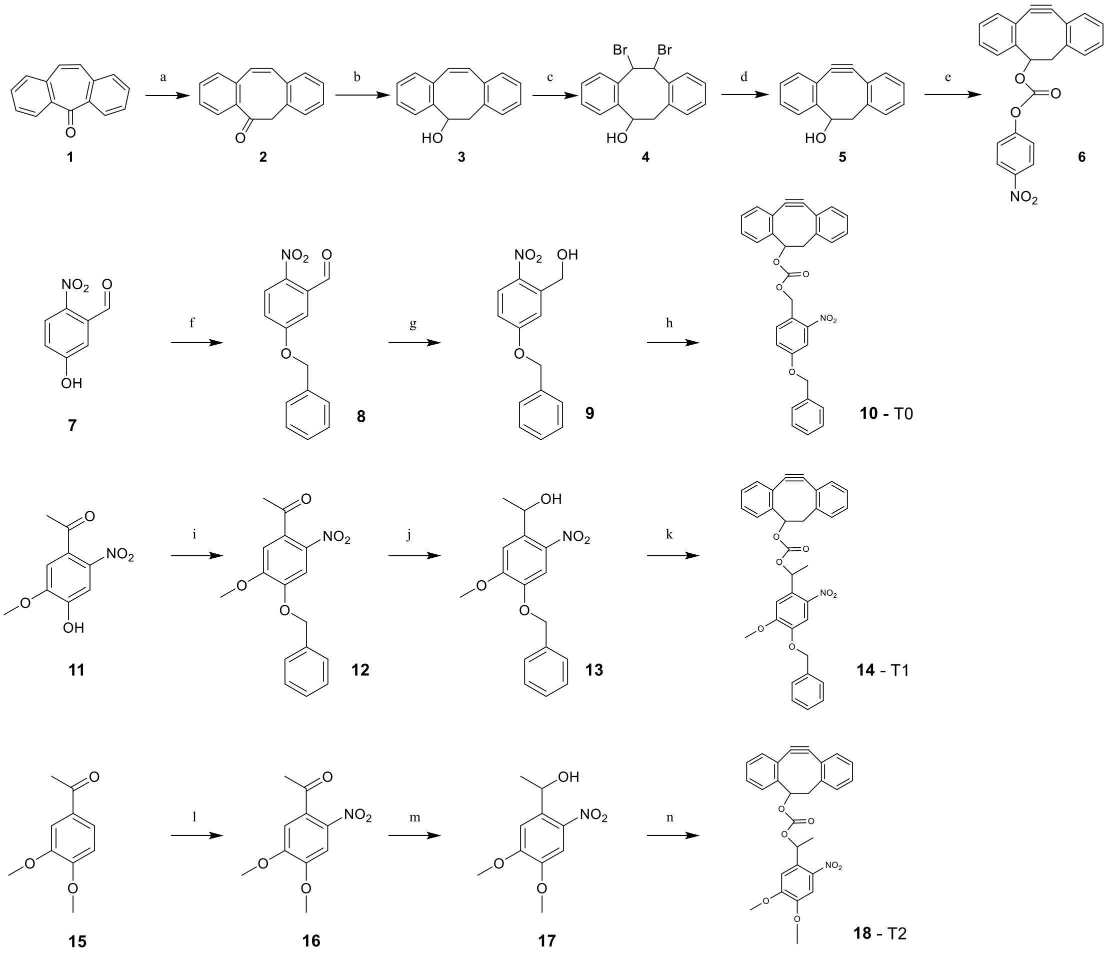

Scheme 1. Reagents and conditions: (a) $TMSCHN_2$, $BF_3{\cdot}OEt_2$, DCM, -10°C, 3 h; (b) $NaBH_4$, EtOH/THF, 7 h; (c) Br_2, $CHCl_3$, 0.5 h; (d) LDA, THF, 0.5 h; (e) 4-nitrophenyl chloroformate, pyridine, DCM, 18 h; (f) potassium carbonate, benzyl bromide, DMF, 10°C, 2h; (g) $NaBH_4$, MeOH, 0°C, 4h; (h) DMAP, DCM, overnight; (i) potassium carbonate, benzyl bromide, DMF, 10°C, 2h; (j) $NaBH_4$, MeOH, 0°C, 4h; (k) DMAP, DCM, overnight; (l) 61% nitric acid, 0°C (m) $NaBH_4$, MeOH, 0°C, 4h; (n) DMAP, DCM, overnight.

Synthesis of Compound 2 (*6H-Dibenzo[a,e]cyclooctatrien-5-one)*

A solution of trimethylsilyl diazomethane (10.5 mL, 21.9 mmol) in CH_2Cl_2 (20 mL) was added dropwise to a stirred solution of dibenzosuberenone (2.88 g, 14.0 mmol) and $BF_3{\cdot}OEt_2$ (2.59 mL, 21.0 mmol) in CH_2Cl_2 (30 mL) at -10 °C over 1 h. The reaction mixture was stirred at -10 °C for 2 h, and then poured into ice water. The aqueous layer was extracted with CH_2Cl_2 (3 × 100 mL) and the combined organic layers washed with brine, dried (Na_2SO_4), filtered and the filtrate was concentrated under reduced pressure. The crude product was purified by silica gel column chromatography (Petroleum ether / ethyl acetate, 40:1-30:1, v/v) to afford **compound 2** as a white solid (2.10 g, 70 %). R_f = 0.50 (Petroleum ether: ethyl acetate = 4:1). ^1H NMR (400 MHz, $CDCl_3$): δ 8.25 (br d, 1H, J = 8.0 Hz), 7.50 - 7.20 (m, 7H), 7.05 (d, 1H, J = 12.9 Hz), 7.02 (d, 1H, J = 12.9 Hz), 4.05 (s, 2H); ^{13}C NMR (100 MHz, $CDCl_3$): δ 196.52, 136.82, 136.20, 135.38, 133.76, 133.09, 132.37, 131.35, 130.53, 129.20, 128.76, 127.91, 127.23, 126.82, 48.38. ESI-HRMS Calcd for $C_{16}H_{12}NaO^+$ $[M+Na]^+$: 243.0780. Found 243.0767.

Synthesis of Compound 3 (*5,6-Dihydro-dibenzo[a,e]cycloocten-5-ol)*

Sodium borohydride (0.76 g, 20 mmol) was slowly added to a stirred solution of **compound 2** (2.20 g, 10 mmol) in a mixture of EtOH and THF (1:1, *v*/*v*, 120 mL). The reaction mixture was stirred for 7 h, after which TLC analysis indicated completion of the reaction. The reaction was quenched by slow addition of acetic acid (1 mL) and the solvents were evaporated. The residue was dissolved in CH_2Cl_2 (100 mL) and the resulting solution was washed with brine (100 mL), which was back extracted with CH_2Cl_2 (4 × 100 mL). The combined organic phases were dried (Na_2SO_4), filtered and concentrated under reduced pressure. The crude product was purified by silica gel column chromatography (Petroleum ether / ethyl acetate, 15:1-2:1, v/v) to give **compound 3** as a white solid (2.0 g, 90%). R_f = 0.30 (Petroleum ether: ethyl acetate = 4:1). ^1H NMR (400 MHz, $CDCl_3$): δ 7.49 - 7.43 (m, 1H), 7.25 - 7.06 (m, 7H), 6.87 (d, 1 H, J = 12.2 Hz), 6.82 (d, 1 H, J = 12.2 Hz), 5.28 (dt, 1H, J = 5.8, 10.4 Hz), 3.53 - 3.25 (m, 2H); ^{13}C NMR (100 MHz, $CDCl_3$): δ 140.76, 136.82, 136.18, 134.46, 131.61, 131.54, 130.15, 129.88, 129.34, 128.63, 127.39, 127.14,

126.96, 125.91, 74.53, 42.55; ESI-HRMS Calcd for $C_{16}H_{14}NaO^+$ $[M+Na]^+$: 245.0937. Found 245.0949.

Synthesis of Compound 4

(*11,12-Dibromo-5,6,11,12-tetrahydrodibenzo[a,e]cycloocten-5-ol*

Bromine (0.51 mL, 10 mmol) as added dropwise to a stirred solution of **compound 3** (2.0 g, 9 mmol) in $CHCl_3$ (50 mL). After stirring the mixture for 0.5 h, TLC analysis indicated completion of the reaction. The solvent was evaporated under reduced pressure to give **Compound 4** as a light-yellow oil, which was directly used in the next step without further purification. R_f = 0.40 (Petroleum ether: CH_2Cl_2 = 2:1).

Compound 5 (DIBO-5) Synthesis of

5,6-Dihydro-11,12-didehydro-dibenzo[a,e]cycloocten-5-ol

Lithium diisopropylamide in THF (2.0 M) (8 mL, 16 mmol) was added dropwise to a stirred solution of **compound 4** (1.3 g, 4.0 mmol) in THF (40 mL). The reaction mixture was stirred for 2 h, after which it was quenched by the dropwise addition of water (0.5 mL). The solvents were evaporated under reduced pressure, and the residue was purified by silica gel column chromatography (Petroleum ether / ethyl acetate, 20:1-10:1, v/v) to to give **compound 5** as a white solid (500 mg, 65%). R_f = 0.50 (Petroleum ether: ethyl acetate = 4:1). ^{1}H NMR (400 MHz, $CDCl_3$): δ 7.74 (d, 1H, J = 7.8 Hz), 7.44 - 7.28 (m, 7H), 4.63 (br s, 1H), 3.10 (dd, 1H, J = 2.2, 14.7 Hz), 2.93 (dd, 1H, J = 3.7, 14.7 Hz), ; ^{13}C NMR (100 MHz, $CDCl_3$): δ 155.55, 151.62, 129.62, 128.05, 127.98, 126.97, 126.83, 126.08, 126.07, 124.03, 123.75, 121.21, 112.90, 110.60, 75.23, 48.70; ESI-HRMS Calcd for $C_{16}H_{12}NaO^+$ $[M+Na]^+$: 243.0786. Found 243.1162.

Synthesis of Compound 6 (*Carbonic acid,*

5,6-dihydro-11,12-didehydro-dibenzo[a,e]cycloocten-5-yl ester, 4-nitrophenyl ester)

4-Nitrophenyl chloroformate (0.40 g, 2 mmol) and pyridine (0.4 mL, 5 mmol) were added to a solution of **compound 5** (0.22 g, 1 mmol) in CH_2Cl_2 (30 mL). After being stirred for 4 h at room temperature, the mixture was washed with brine (2 ×10 mL) and the organic layer was dried (Na_2SO_4). The solvents were evaporated under reduced pressure, and the residue was purified by silica gel column chromatography (Petroleum ether /ethyl acetate, 10:1, v/v) to afford **compound 6** (0.34 g, 89%). R_f = 0.65 (Petroleum ether: ethyl acetate = 4:1). ^{1}H NMR (400 MHz, $CDCl_3$): δ 8.34 - 8.23 (2H, aromatics), 7.67 - 7.59 (1H, aromatics), 7.45 - 7.31 (9H, aromatics), 5.59 (br s,

1H), 3.34 (dd, J = 2.1, 15.4 Hz, 1H), 3.05 (dd, 1H, J = 4.0, 15.4 Hz); ^{13}C NMR (100 MHz, $CDCl_3$): δ 155.44, 151.69, 150.12, 149.71, 145.38, 129.97, 128.31, 128.25, 127.73, 127.46, 126.51, 126.14, 125.31, 123.60, 123.44, 121.65, 121.20, 113.17, 109.51, 81.58, 45.75; ESI-HRMS Calcd for $C_{23}H_{15}NNaO_5^+$ $[M+Na]^+$: 408.0842. Found 408.0852.

Synthesis of Compound 8 (*5-(benzyloxy)-2-nitrobenzaldehyde)*

To a stirred solution of **compound 7** (1 g, 6 mmol) in DMF (10 mL) were added potassium carbonate (1 g, 7 mmol) and benzyl bromide (1 mL, 6.6 mmol) at room temperature and the mixture was stirred for 2 h at 45°C. After cooling, the mixture was diluted with EtOAc, washed with H_2O and brine, dried with Na_2SO_4, and concentrated in vacuo to provide **compound 8** as a yellow solid (1.2 g, 90%). R_f = 0.80 (Petroleum ether: ethyl acetate = 1:1). ^{1}H NMR (400 MHz, $CDCl_3$): δ 10.47 (s, 1H), 8.16 (d, 1H, J = 9.0 Hz), 7.44 - 7.35 (m, 6H), 7.21 (dd, 1H, J = 2.8, 9.0 Hz), 5.21 (s, 2H); ^{13}C NMR (100 MHz, $CDCl_3$): δ 188.39, 163.06, 142.41, 134.92, 134.30, 128.85, 128.68, 127.57, 127.27, 119.29, 114.21, 71.06.

Synthesis of Compound 9 *(5-(benzyloxy)-2-nitrophenyl) methanol*

Compound 8 (200 mg, 1 mmol) was then dissolved in methanol (10 mL) at 0°C, and $NaBH_4$ (80 mg, 4 mmol) was added. After stirring at room temperature for 1 h, the solution was concentrated under reduced pressure and the residue taken up with DCM (20 mL × 3). The organic layer was dried over sodium sulfate, and and concentrated in vacuo to provide **compound 9** (200 mg, 100%). R_f = 0.70 (Petroleum ether: ethyl acetate = 1:1). ^{1}H NMR (400 MHz, $CDCl_3$): δ 8.16 (d, 1H, J = 9.1 Hz), 7.48 - 7.33 (m, 5H), 7.32 (d, 1H, J = 2.8 Hz), 6.95 (dd, 1H, J = 1.8, 9.1 Hz), 5.17 (s, 2H), 4.98 (d, 1H, J = 5.8 Hz); ^{13}C NMR (100 MHz, $CDCl_3$): δ 163.29, 140.42, 140.33, 135.49, 128.77, 128.47, 127.98, 127.50, 115.02, 113.88, 70.62, 62.89.

Synthesis of Synthesis of Compound 10 *(Carbonic acid, 5,6-dihydro-11,12-didehydro-dibenzo[a,e]cycloocten-5-yl ester, 4-(benzyloxy)-2-nitrobenzyl ester)*

To a solution of **compound 6** (50 mg, 0.13 mmol) and **compound 9** (60 mg, 0.20 mmol) in 10 mL DCM was added DMAP (29 mg, 0.20 mmol). The reaction was stirred at room temperature overnight. The solvents were evaporated under reduced pressure, and the residue was purified by silica gel column chromatography (Petroleum ether /ethyl acetate, 10:1, v/v) to afford **compound 10** (35 mg, 54%). R_f = 0.50 (Petroleum ether: ethyl acetate = 4:1) ^{1}H NMR ($CDCl_3$, 400 MHz): δ 8.22 (d, 1H,

J = 9.1 Hz, aromatics), 7.59 (d, 1H, J = 7.8 Hz, aromatics), 7.47 - 7.20 (m, 14H, aromatics), 6.98 (d, 1H, J = 9.0 Hz, aromatics), 5.64 (s, 2H), 5.51 (s, 1H), 5.16 (s, 2H), 3.27 (br d, 1H, J = 15.2 Hz), 2.98 (dd, 1H, J = 3.8, 15.2 Hz); ^{13}C NMR ($CDCl_3$, 100 MHz): 163.12, 153.90, 150.47, 150.43, 140.08, 135.29, 135.04, 129.95, 128.82, 128.77, 128.60, 128.21, 128.15, 128.12, 127.62, 127.47, 127.28, 126.35, 126.03, 123.65, 123.62, 121.21, 114.44, 113.60, 113.10, 109.65, 80.56, 70.76, 66.60, 45.92, 26.90; ESI-HRMS Calcd for $C_{31}H_{27}N_2O_6$ $[M+NH_4]^+$: 523.1869. Found 523.1875.

Synthesis of Compound 12 ***(1-(4-(benzyloxy)-5-methoxy-2-nitrophenyl)ethan-1-one)***

To a stirred solution of **compound 11** (1 g, 6 mmol) in DMF (10 mL) were added potassium carbonate (1.25 g, 9 mmol) and benzyl bromide (1 mL, 6.6 mmol) at room temperature and the mixture was stirred for 2 h at 45°C. After cooling, the mixture was diluted with EtOAc, washed with H_2O and brine, dried with Na_2SO_4, and concentrated in vacuo to provide **compound 12** as a yellow solid (1.2 g, 90%). R_f = 0.60 (Petroleum ether: ethyl acetate = 1:1). ^{1}H NMR (400 MHz, $CDCl_3$): δ 7.67 (s, 1H), 7.48 - 7.30 (m, 6H), 6.77 (s, 1H), 5.22 (s, 2H), 3.98 (s, 3H), 2.49 (s, 3H); ^{13}C NMR (100 MHz, $CDCl_3$): δ 200.01, 154.54, 148.57, 138.28, 135.21, 133.08, 128.82, 128.55, 127.54, 108.85, 108.84, 71.41, 56.65, 30.35.

Synthesis of Compound 13 ***(1-(4-(benzyloxy)-5-methoxy-2-nitrophenyl)ethan-1-ol)***

Compound 12 (200 mg, 1 mmol) was dissolved in methanol (10 mL), and $NaBH_4$ (80 mg, 4 mmol) was added slowly. After stirring at room temperature for 1 h, the solution was concentrated under reduced pressure and the residue taken up with DCM (20 mL × 3). The organic layer was dried over sodium sulfate, and and concentrated in vacuo to provide **compound 13** (200 mg, 100%). R_f = 0.50 (Petroleum ether: ethyl acetate = 1:1). ^{1}H NMR (400 MHz, $CDCl_3$): δ 7.62 (s, 1H), 7.54 - 7.28 (m, 6H), 5.54 (q, 1H, J = 6.2 Hz), 5.17 (s, 2H), 3.99 (s, 3H), 1.54 (d, 3H, J = 6.3 Hz); ^{13}C NMR (100 MHz, $CDCl_3$): δ 154.29, 146.63, 139.41, 137.11, 135.67, 128.71, 128.33, 127.53, 109.76, 108.72, 71.20, 65.74, 56.37, 24.21.

Synthesis of Compound 14 ***(Carbonic acid, 5,6-dihydro-11,12-didehydro-dibenzo[a,e]cycloocten-5-yl ester, 1-(4-(benzyloxy)-5-methoxy-2-nitrophenyl)ethyl ester)***

To a solution of **compound 6** (60 mg, 0.16mmol) and **compound 13** (71 mg, 0.23mmol) in 5 mL DCM was added DMAP (29 mg, 0.23 mmol). The reaction was stirred at room temperature overnight. The solvents were evaporated under reduced pressure, and the residue was purified by silica gel column chromatography

(Petroleum ether /ethyl acetate, 10:1, v/v) to afford **compound 14** (43mg, 50%). R_f = 0.45 (Petroleum ether: ethyl acetate = 4:1). ^{1}H NMR ($CDCl_3$, 400 MHz): δ 8.32 (d, J = 8.7 Hz, 2H, aromatics), 7.64 (d, J = 16.2 Hz, 1H, aromatics), 7.52 - 7.24 (m, 11H, aromatics), 7.14 (d, J = 4.6 Hz, 1H, aromatics), 6.43 - 6.34 (m, 1H, -C*H*-CH_3), 5.41 (s, 1H, -C*H*-CH_2-), 5.17, 5.13 (s, 2H, -OCH_2-), 4.01, 3.99 (s, 3H, -OCH_3), 3.26 - 2.88 (m, 2H, -CH-C*H_2*-), 1.75 - 1.67 (m, 3H, -CH-C*H_3*); ^{13}C NMR ($CDCl_3$, 100 MHz): 154.79, 154.32, 154.30, 153.18, 153.17, 150.51, 150.44, 150.34, 149.99, 147.25, 147.19, 145.86, 139.77, 139.62, 135.54, 132.97, 132.61, 129.89, 129.69, 128.74, 128.71, 128.39, 128.36, 128.23, 128.13, 128.07, 127.82, 127.53, 127.44, 127.29, 126.39, 126.35, 126.02, 125.48, 123.65, 123.57, 123.45, 123.21, 121.60, 113.12, 113.01, 109.72, 109.53, 108.13, 107.80, 80.31, 80.23, 72.52, 72.45, 71.26, 71.24, 56.52, 56.42, 45.89, 45.67, 21.98, 21.92; ESI-HRMS Calcd for $C_{33}H_{31}N_2O_7$ $[M+NH_4]^+$: 567.2131. Found 567.2135.

Synthesis of Compound 15 ***(1-(4,5-dimethoxy-2-nitrophenyl)ethan-1-one)***

Into a recovery flask immersed in a water bath, 61% nitric acid (10 mL) was added. While maintaining the temperature back and forth as 0°C, **compound 14** (2.17 g, 12 mmol) solved in acetone was added in portions**.** The reaction was stirred for 5 h and then poured in 150 mL ice-water. The residue was extracted with CH_2Cl_2 (30 mL × 3) and the obtained organic solution was dried over Na_2SO_4, and concentrated in a vacuum to provide the product as a yellow solid (1.63 g, 50%). R_f = 0.30 (Petroleum ether: ethyl acetate = 2:1). ^{1}H NMR ($CDCl_3$, 400 MHz): δ 7.61 (s, 1H), 6.76 (s, 1H), 3.98 (s, 6H), 2.50 (s, 3H); ^{13}C NMR ($CDCl_3$, 100 MHz): 200.04, 153.96, 149.57, 138.44, 132.83, 108.56, 106.79, 56.67, 56.57, 30.39.

Synthesis of Compound 17 ***(1-(4,5-dimethoxy-2-nitrophenyl)ethan-1-ol)***

To a solution of **compound 16** (100 mg) in 10 mL methanol was added $NaBH_4$ (0.26 mg, 6.8 mmol). The reaction was stirred at room temperature for 4 h and acidified with 1 N HCl to pH = 6. After concentration under reduced pressure to remove the methanol, the residue was extracted with CH_2Cl_2 (30 mL × 3) and the obtained organic phase was dried over Na_2SO_4 and concentrated in a vacuum to provide the product as a yellow solid (90 mg, 90%). R_f = 0.20 (Petroleum ether: ethyl acetate = 2:1) ^{1}H NMR ($CDCl_3$, 400 MHz): δ 7.55 (s, 1H), 7.29 (s, 1H), 5.56 (q, J = 6.2 Hz, 1H), 3.99 (s, 3H), 3.93 (s, 3H), 1.54 (d, J = 6.2 Hz, 3H); ^{13}C NMR ($CDCl_3$, 100 MHz): 153.71, 147.70, 139.64, 136.80, 108.45, 107.63, 65.76, 56.40, 56.34, 24.26.

Synthesis of compound 17 (*Carbonic acid, 5,6-dihydro-11,12-didehydro-dibenzo[a,e]cycloocten-5-yl ester, 1-(4,5-dimethoxy-2-nitrophenyl)ethyl ester)*

To a solution of **compound 6** (60 mg, 0.16mmol) and **compound 17** (52 mg, 0.23mmol) in 5 mL DCM was added DMAP (29 mg, 0.23 mmol). The reaction was stirred at room temperature overnight. The solvents were evaporated under reduced pressure, and the residue was purified by silica gel column chromatography (Petroleum ether /ethyl acetate, 8:1, v/v) to afford **compound 18** (44 mg, 60%). R_f = 0.45 (Petroleum ether: ethyl acetate = 3:1). ^{1}H NMR ($CDCl_3$, 400 MHz): δ 7.64 - 7.10 (m, 10H, aromatics), 6.50 - 6.31 (m, 1H, -C*H*-CH_3), 5.41 (s, 1H, -C*H*-CH_2), 4.10 - 3.97 (m, 3H, -OCH_3), 3.97 - 3.87 (m, 3H, -OCH_3), 3.28 - 2.88 (m, 2H, -CH-CH_2), 1.83 - 1.66 (m, 3H, -CH-CH_3); ^{13}C NMR ($CDCl_3$, 100 MHz): 153.76, 153.74, 153.21, 153.19, 150.50, 150.44, 150.34, 148.19, 148.11, 139.96, 139.82, 132.78, 132.40, 129.91, 129.66, 128.25, 128.14, 128.10, 127.81, 127.48, 127.32, 126.43, 126.39, 126.08, 126.05, 123.68, 123.58, 123.43, 123.18, 121.31, 121.17, 115.57, 113.13, 113.02, 109.69, 109.53, 107.77, 107.70, 107.45, 80.35, 80.26, 77.00, 72.53, 72.47, 56.55, 56.45, 56.39, 56.35, 45.84, 45.65, 22.02, 21.97; ESI-HRMS Calcd for $C_{27}H_{27}N_2O_7$ $[M+NH_4]^+$: 491.1818. Found 491.1817.

^{1}H NMR of compound **2**

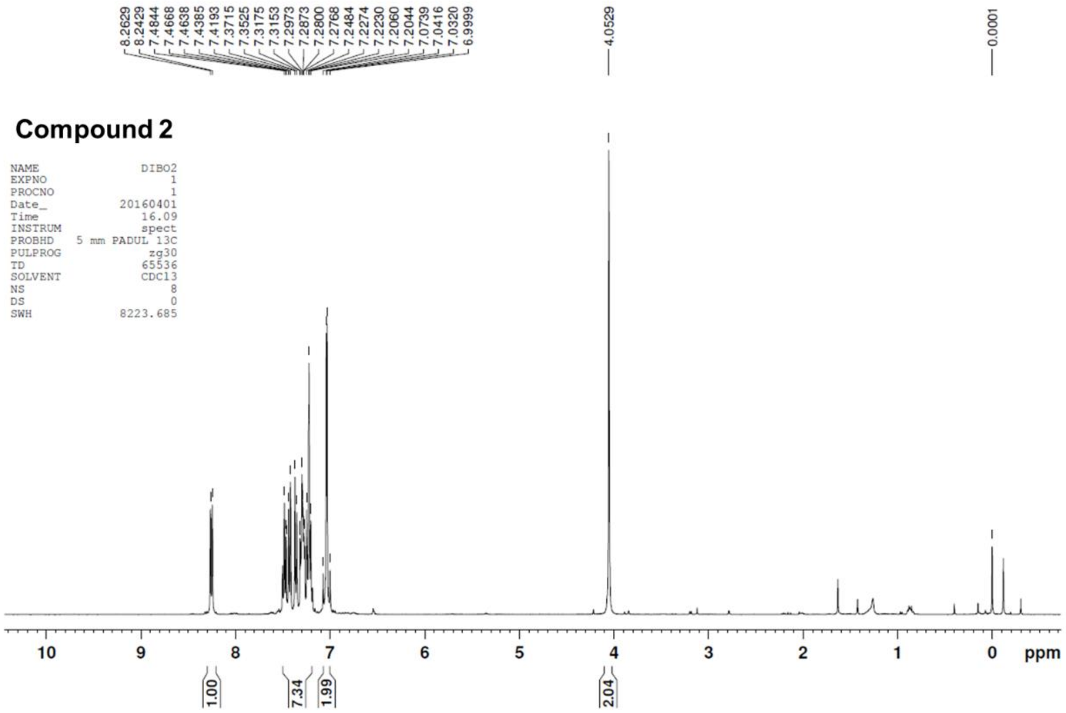

^{13}C NMR of compound **2**

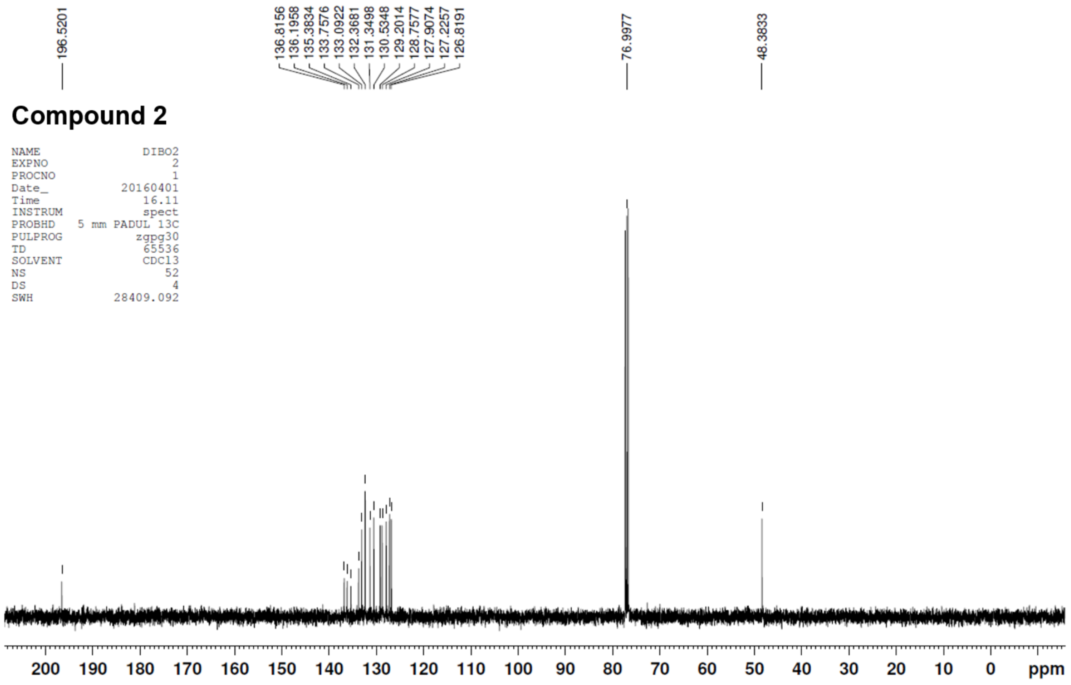

^{1}H NMR of compound **3**

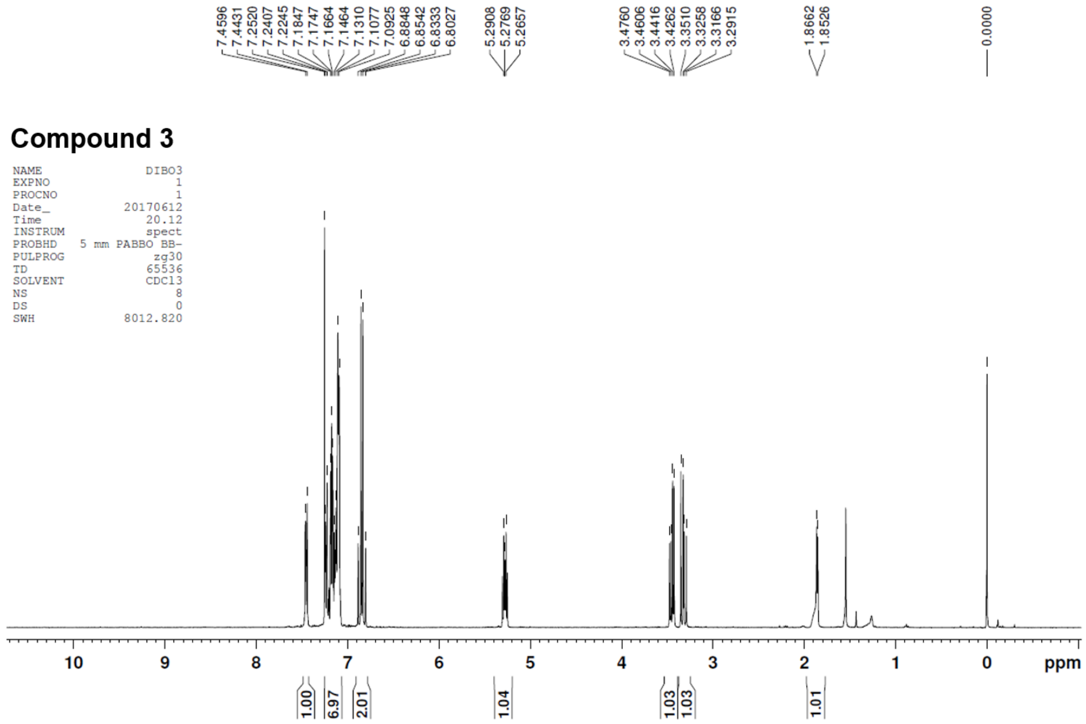

^{13}C NMR of compound **3**

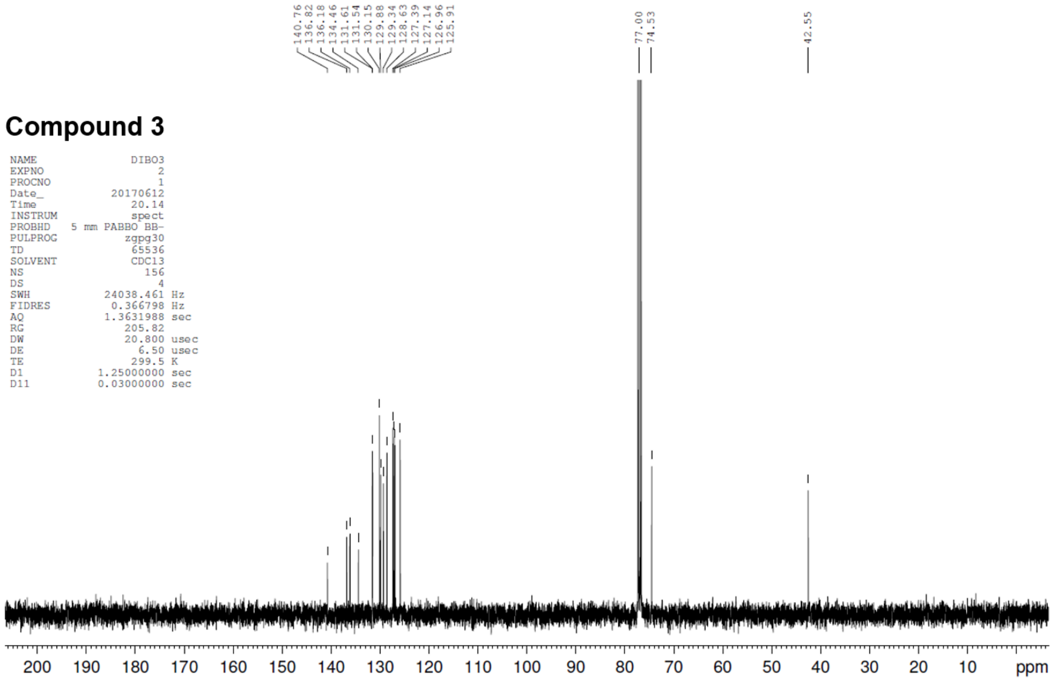

^{1}H NMR of compound **5**

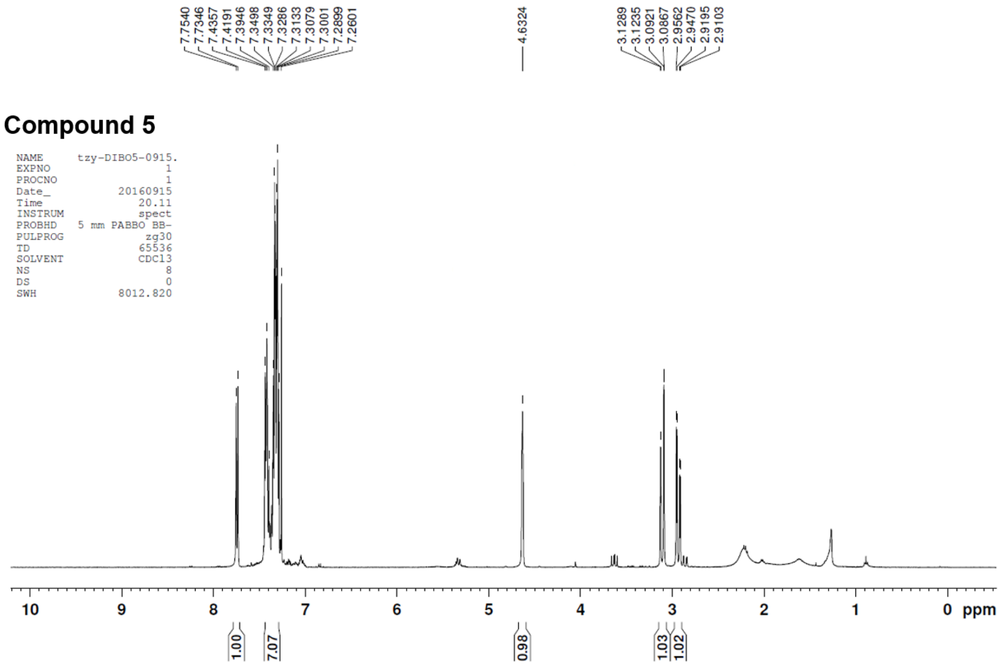

^{13}C NMR of compound **5**

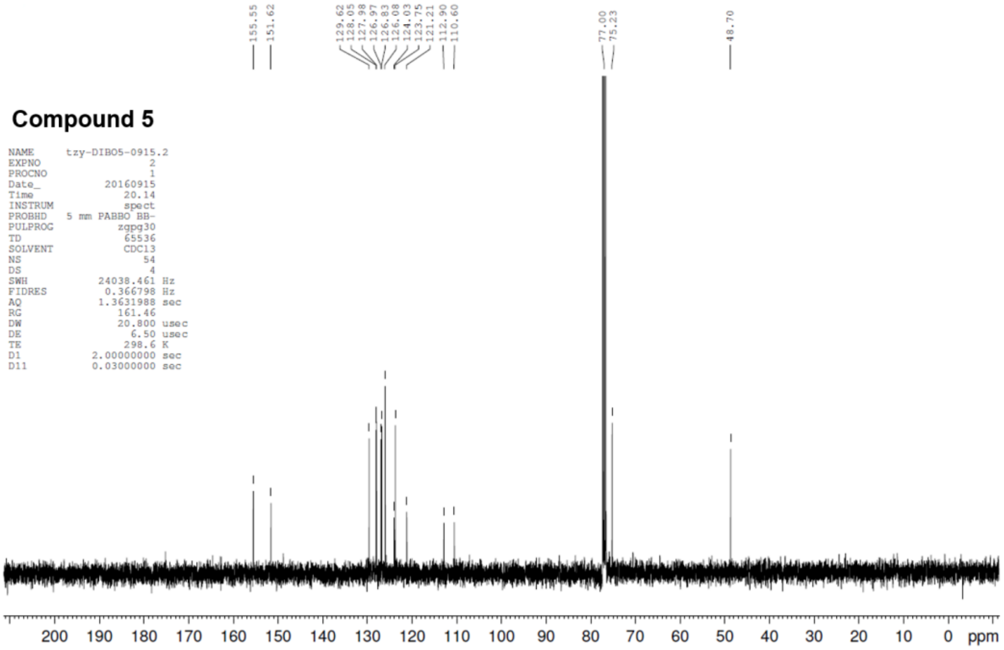

^{1}H NMR of compound **6**

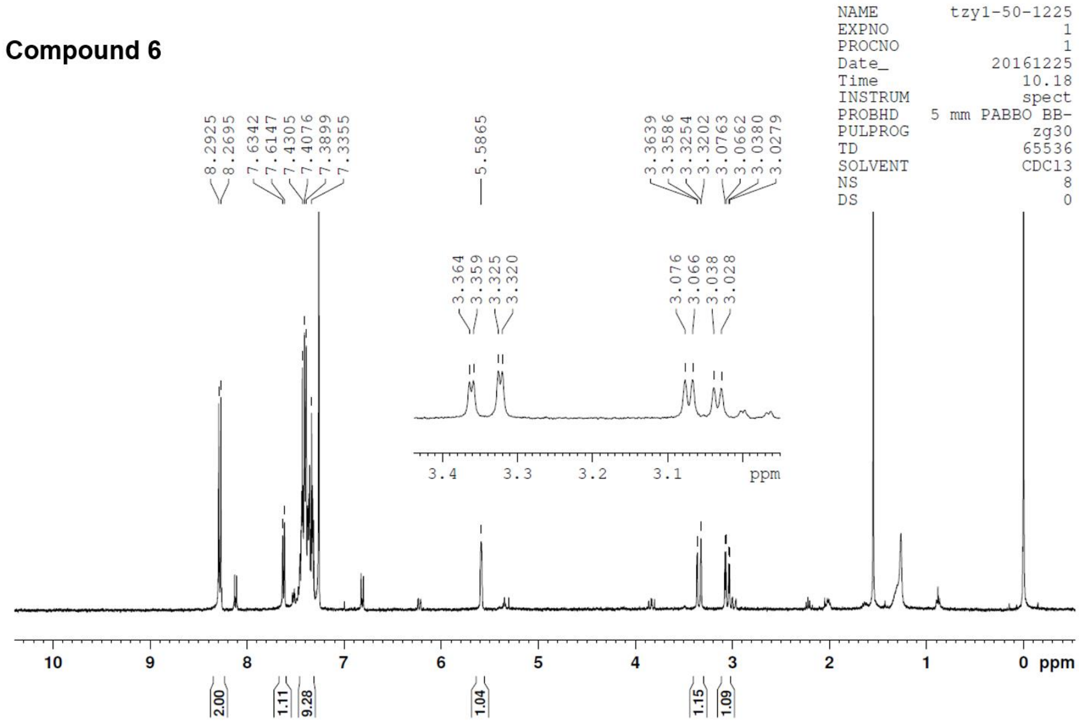

^{13}C NMR of compound **6**

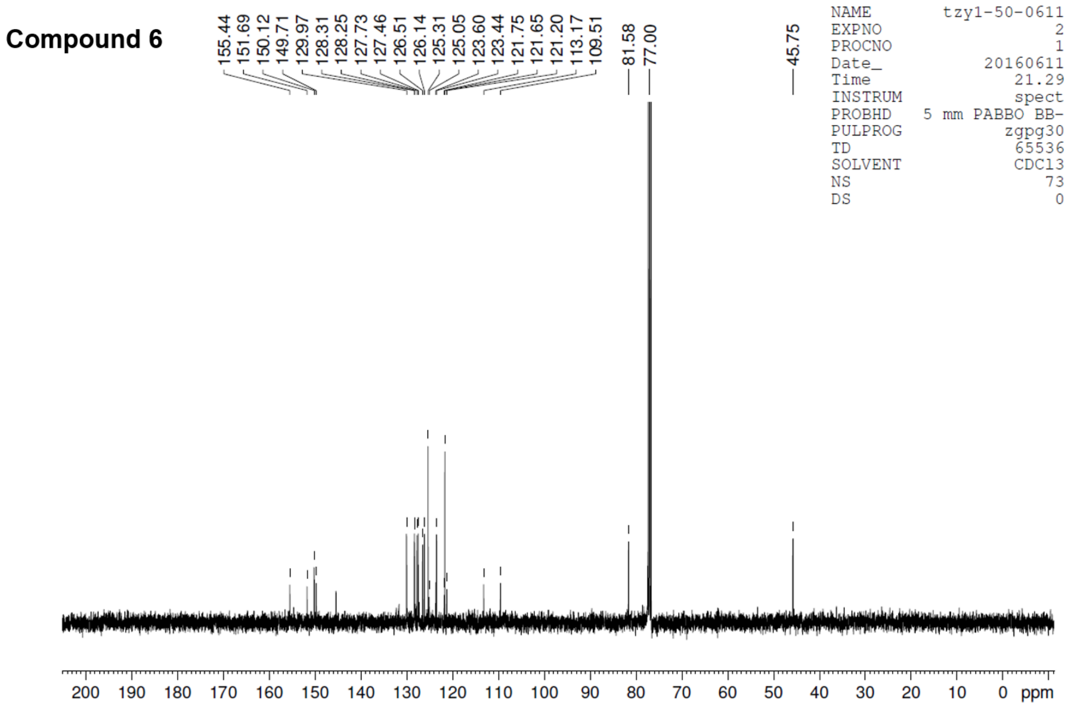

^{1}H NMR of compound **8**

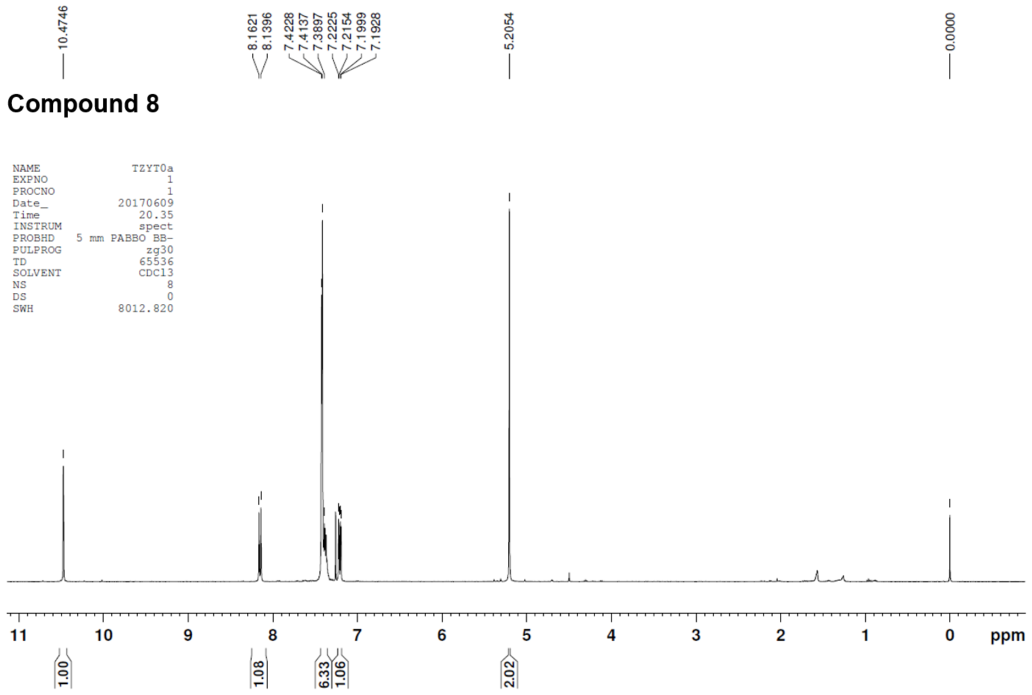

^{13}C NMR of compound **8**

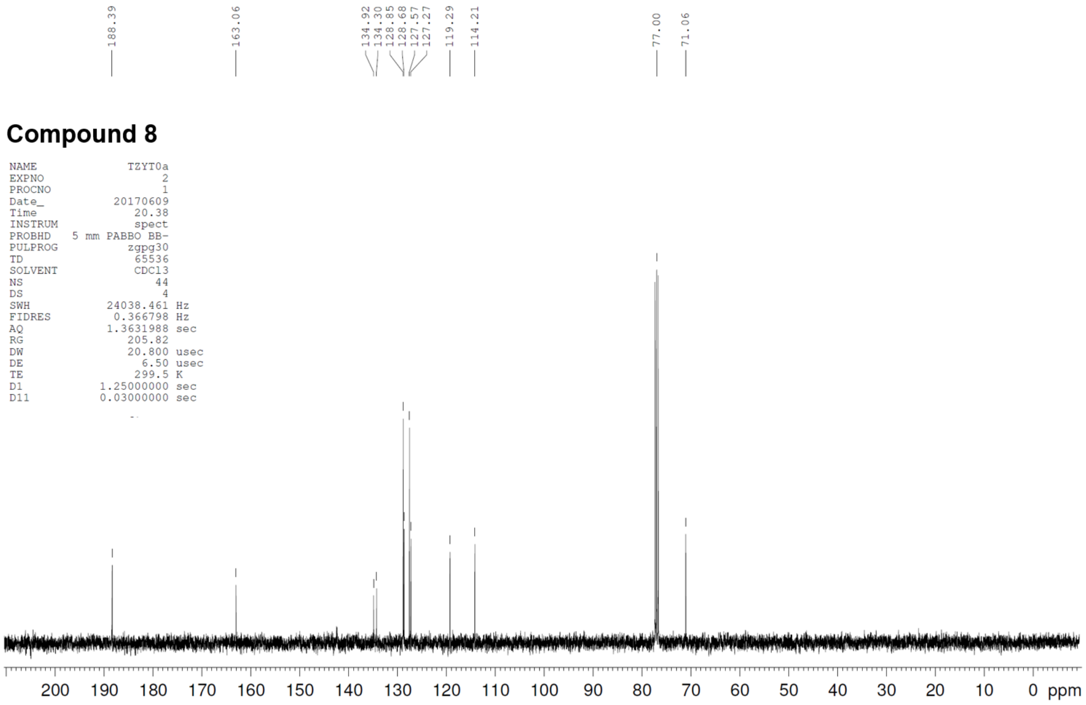

^{1}H NMR of compound **9**

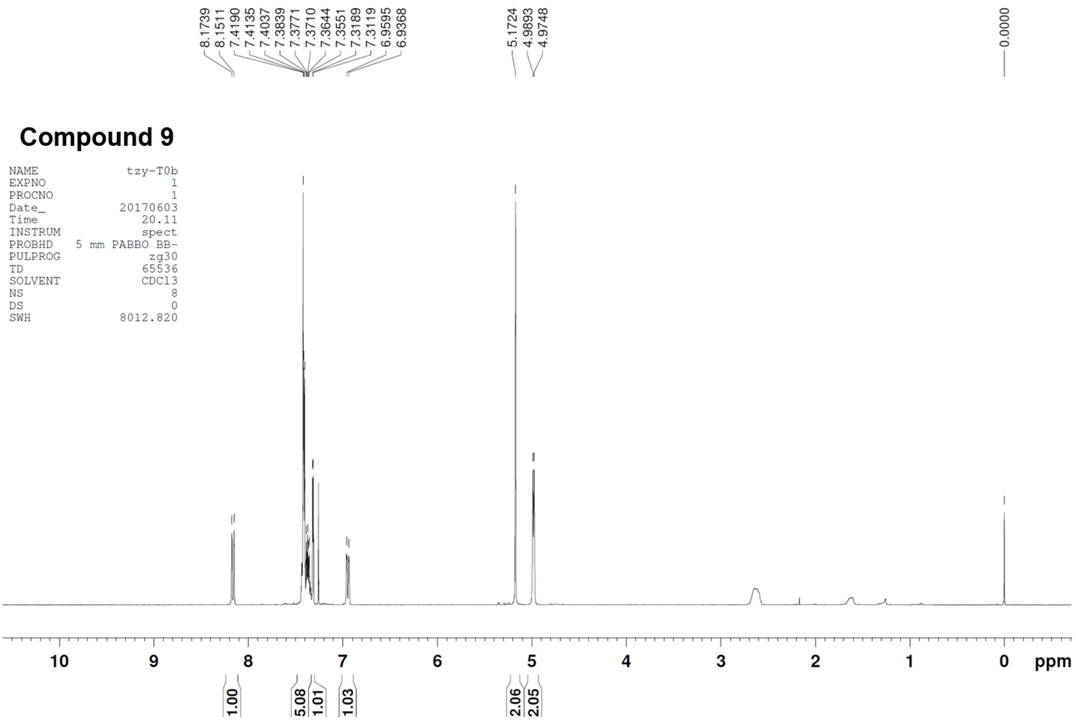

^{13}C NMR of compound **9**

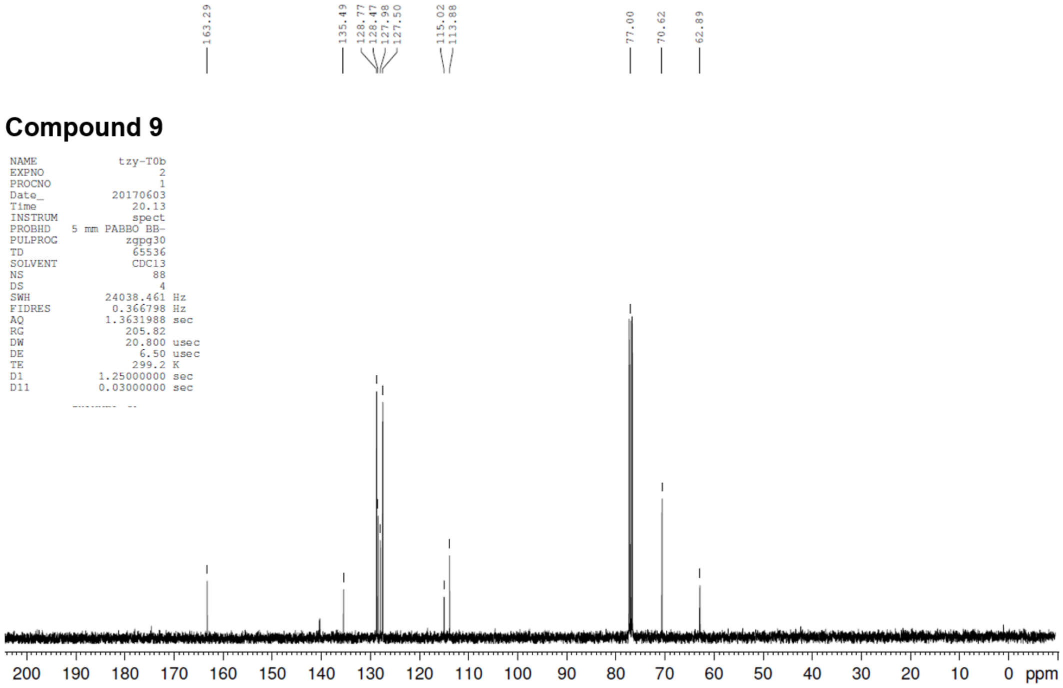

^{1}H NMR of compound **10**

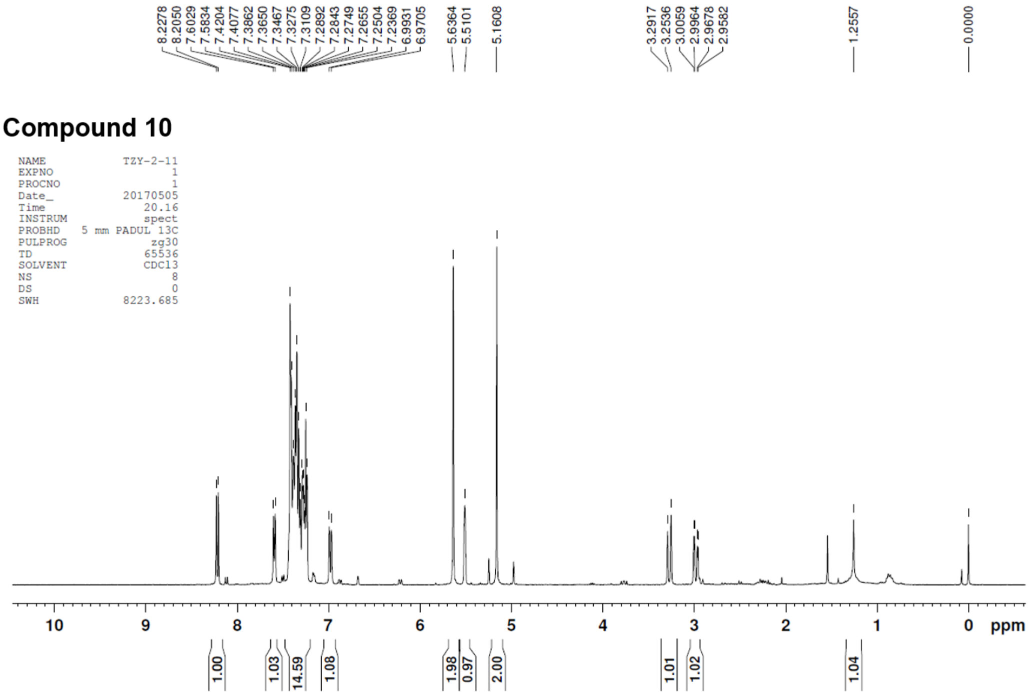

^{13}C NMR of compound **10**

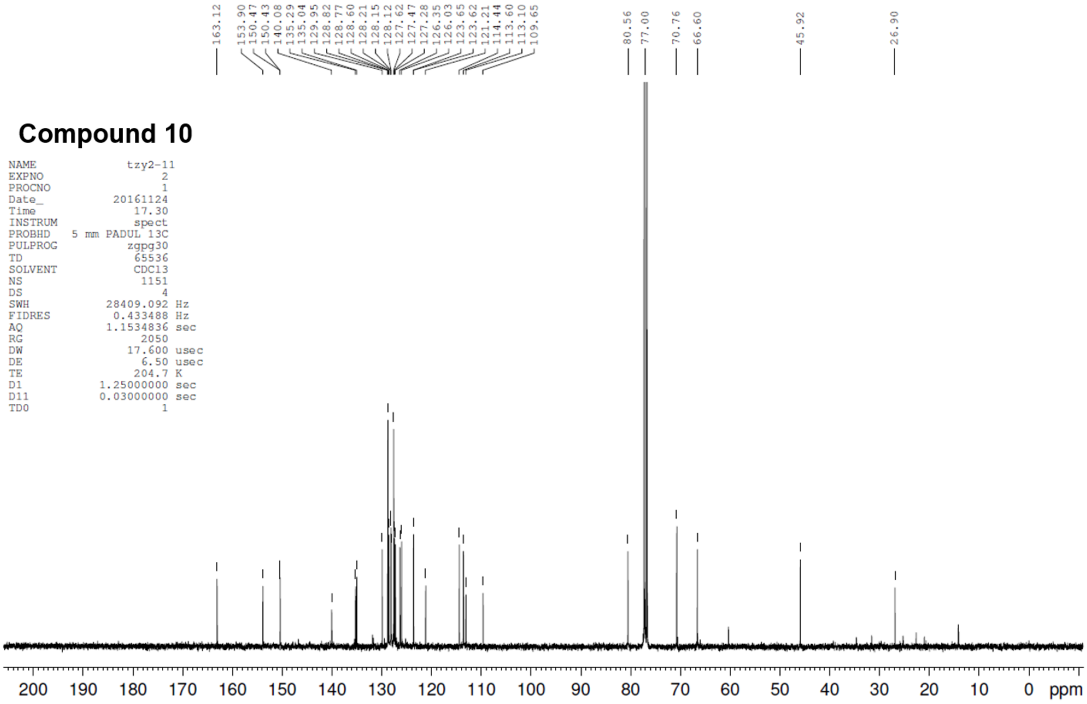

HRMS of compound **10**

Peking University Mass Spectrometry Sample Analysis Report

Analysis Info

Analysis Name	FTMS-17050215_Pos_20170517_000004.d	Acquisition Date	5/17/2017 3:54:24 PM
Sample	**Compound 10**	Instrument	Bruker Solarix XR FTMS
Comment		Operator	Peking University

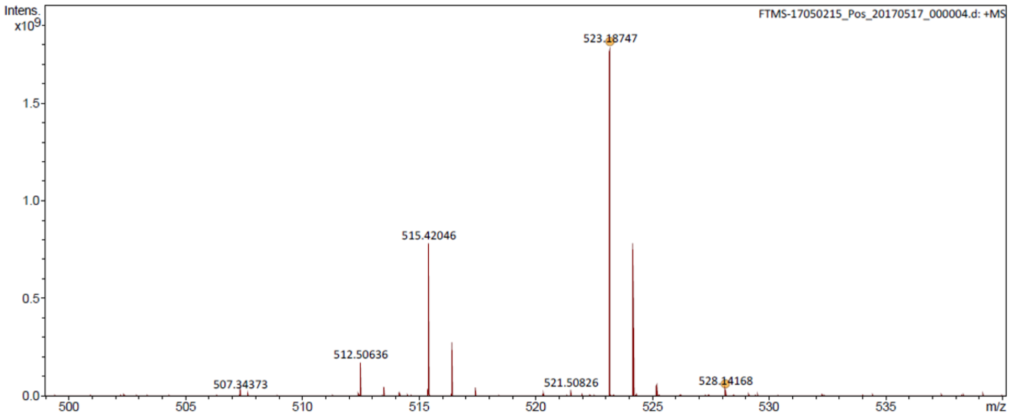

Meas. m/z	#	Ion Formula	Score	m/z	err [ppm]	Mean err [ppm]	mSigma	rdb	e⁻ Conf	N-Rule
523.187469	1	C31H27N2O6	100.00	523.186363	-2.1	-1.3	59.9	20.0	even	ok
528.141682	1	C31H23NNaO6	100.00	528.141758	0.1	-0.2	12.6	21.0	even	ok

^{1}H NMR of compound **12**

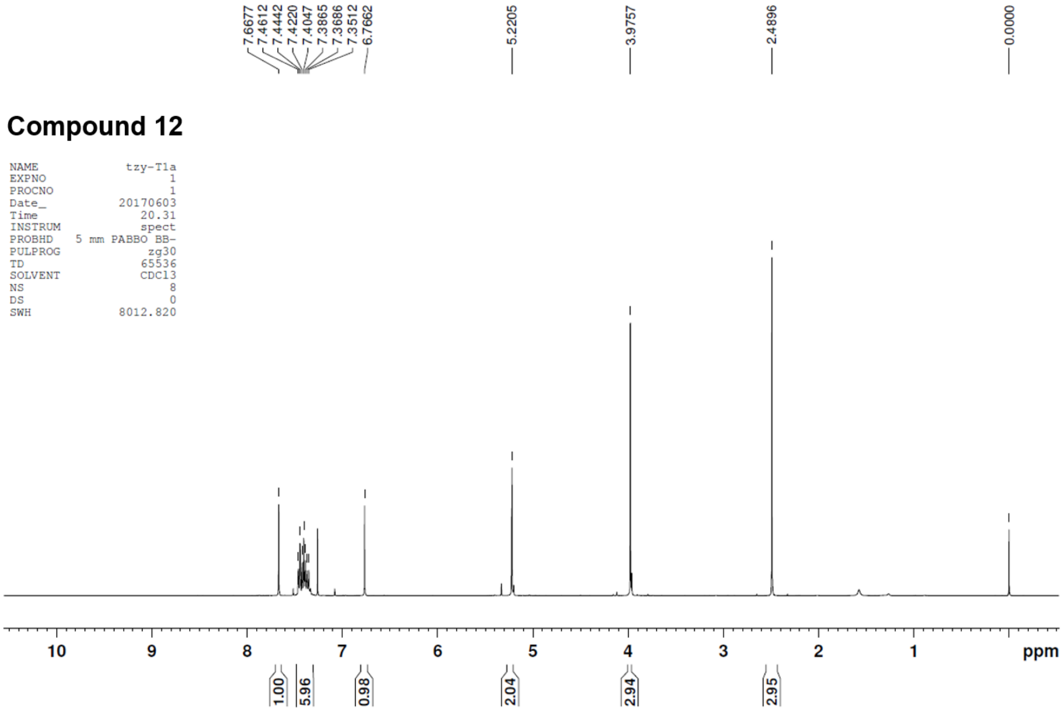

^{13}C NMR of compound **12**

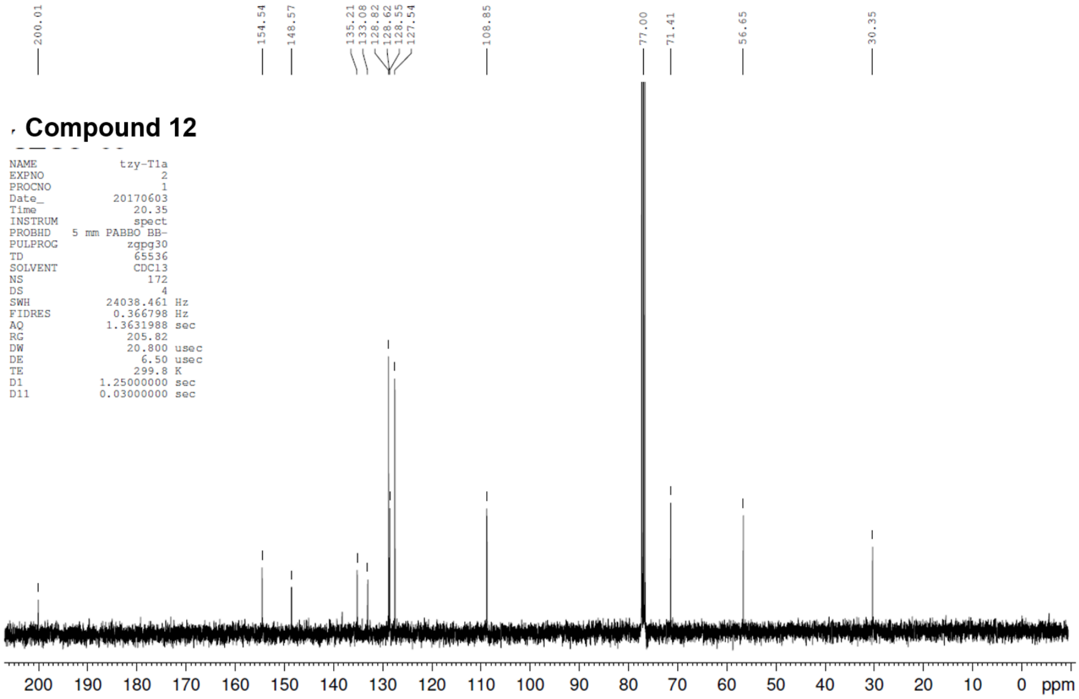

^{1}H NMR of compound **13**

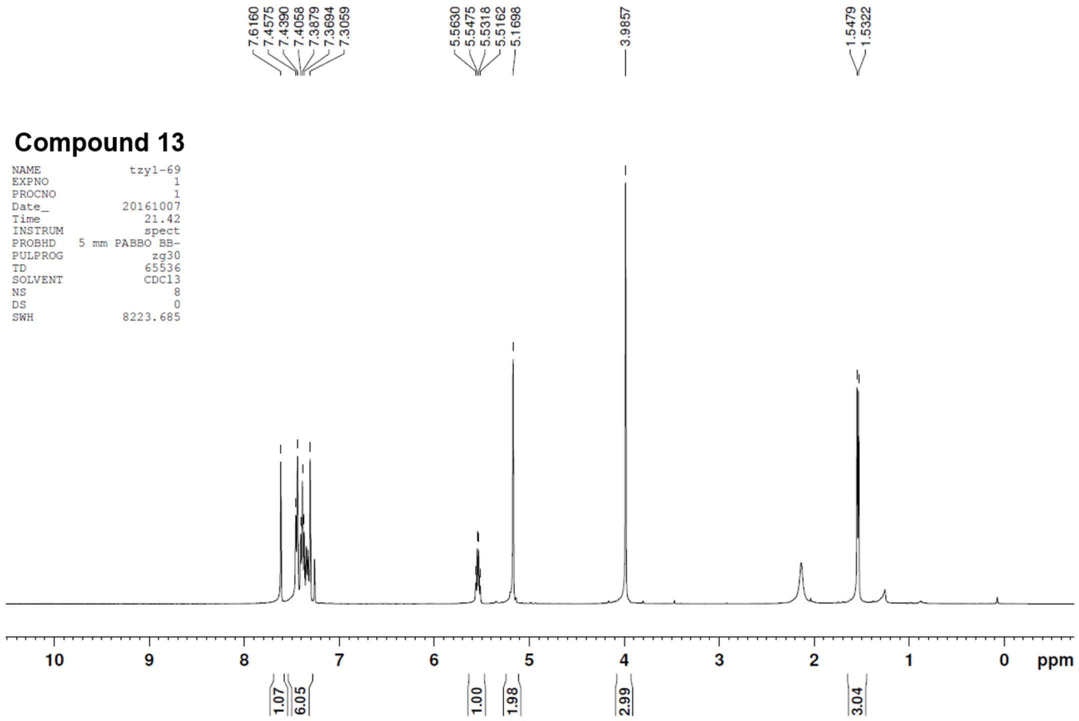

^{13}C NMR of compound **13**

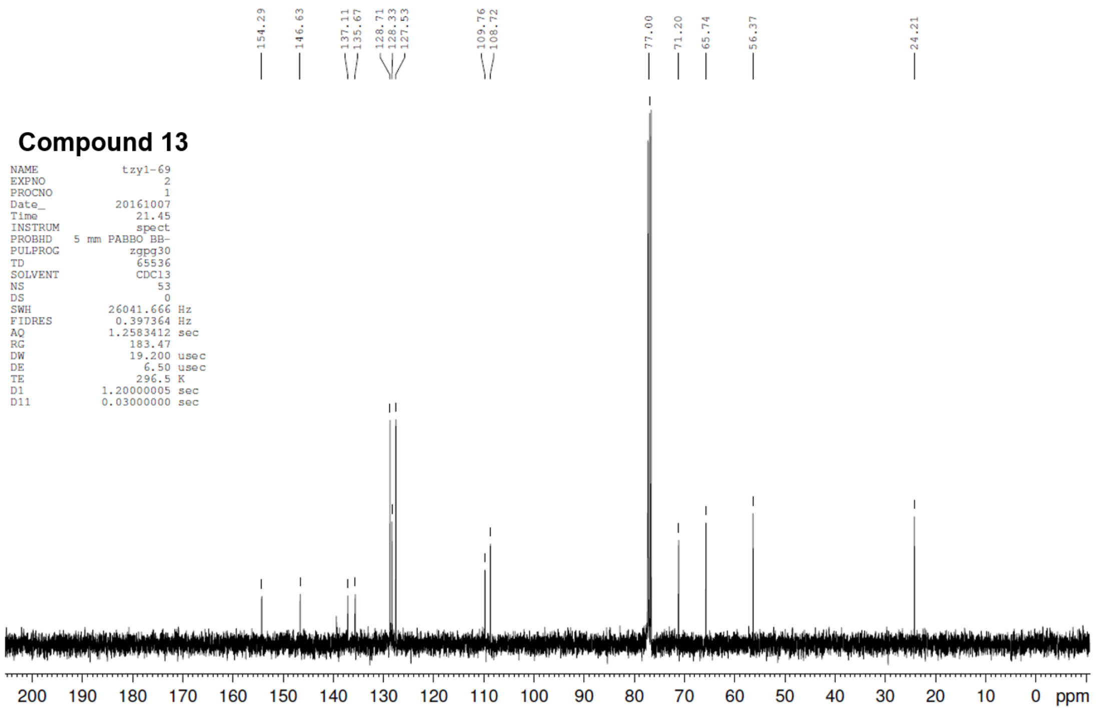

^{1}H NMR of compound **14**

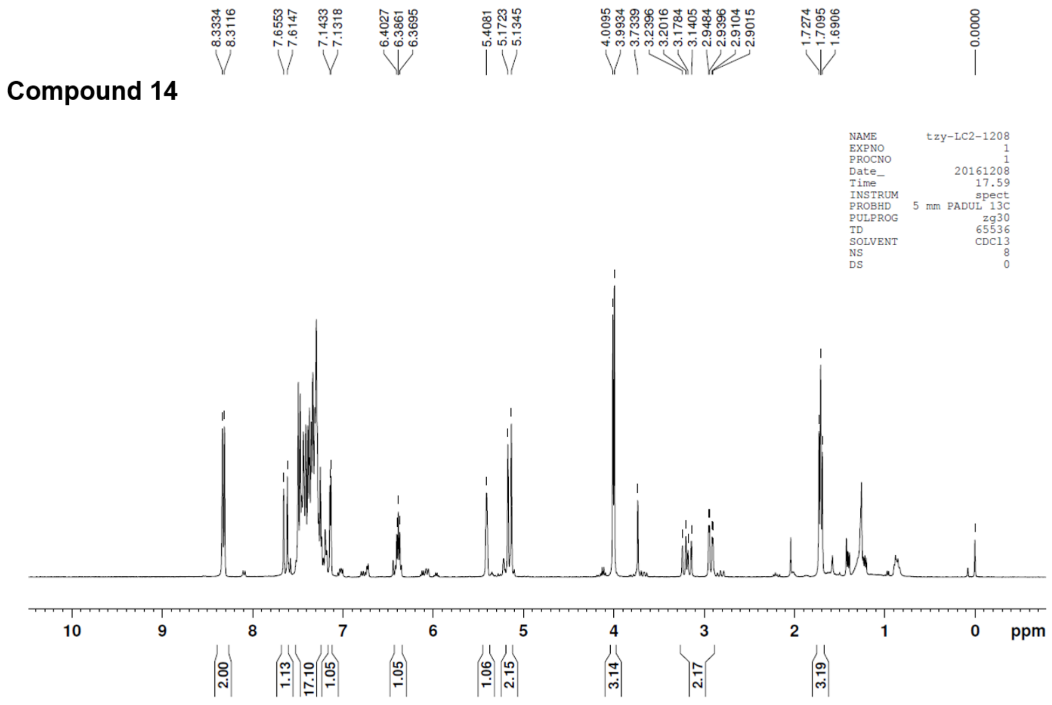

^{13}C NMR of compound **14**

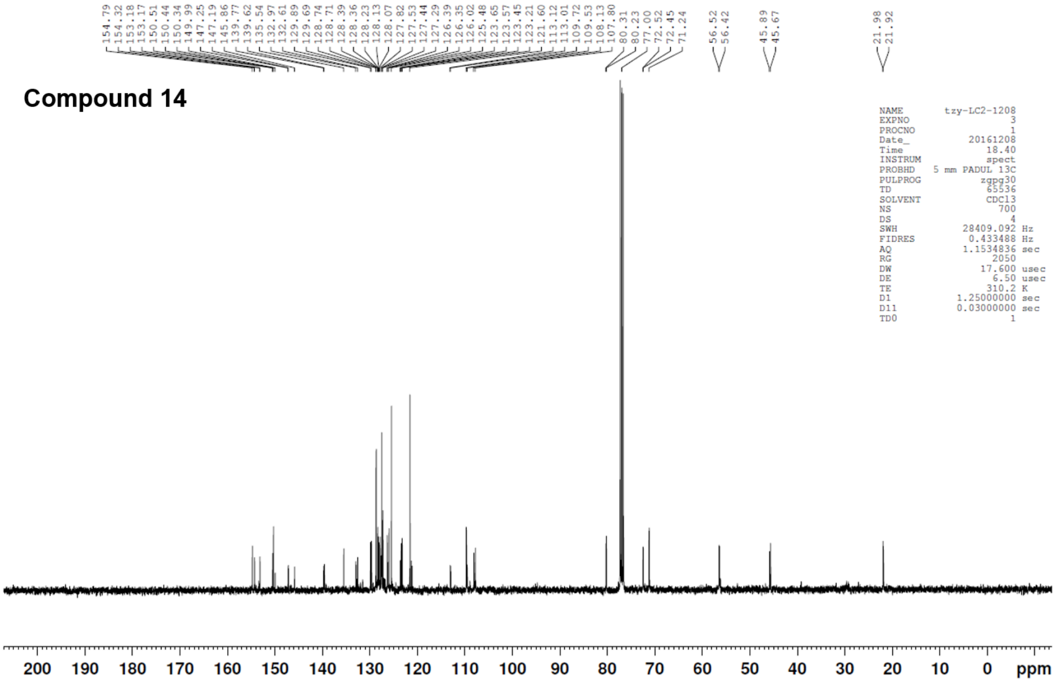

HRMS of compound **14**

Peking University Mass Spectrometry Sample Analysis Report

Analysis Info

Analysis Name	FTMS-17040330_Pos_20170424_000001.d	Acquisition Date	4/25/2017 2:51:09 PM
Sample		Instrument	Bruker Solarix XR FTMS
Comment	**Compound 14**	Operator	Peking University

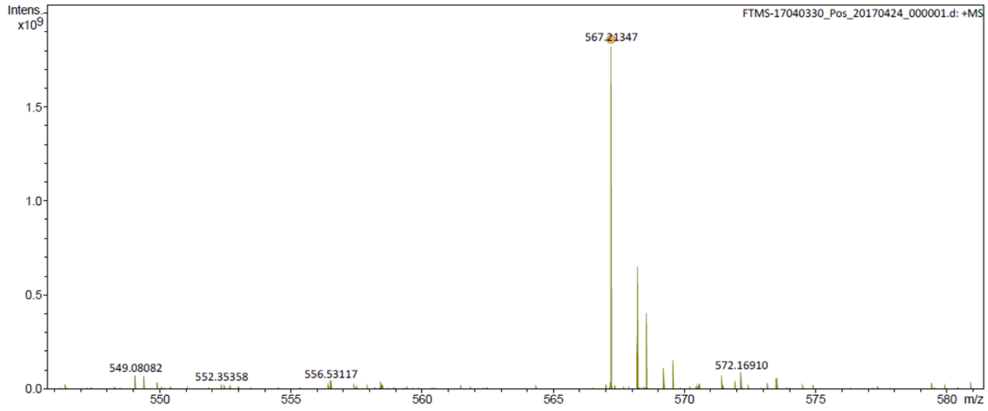

Meas. m/z	#	Ion Formula	Score	m/z	err [ppm]	Mean err [ppm]	mSigma	rdb	e⁻ Conf	N-Rule
567.213473	1	C33H31N2O7	100.00	567.212578	-1.6	-2.5	12.5	20.0	even	ok

^{1}H NMR of compound **16**

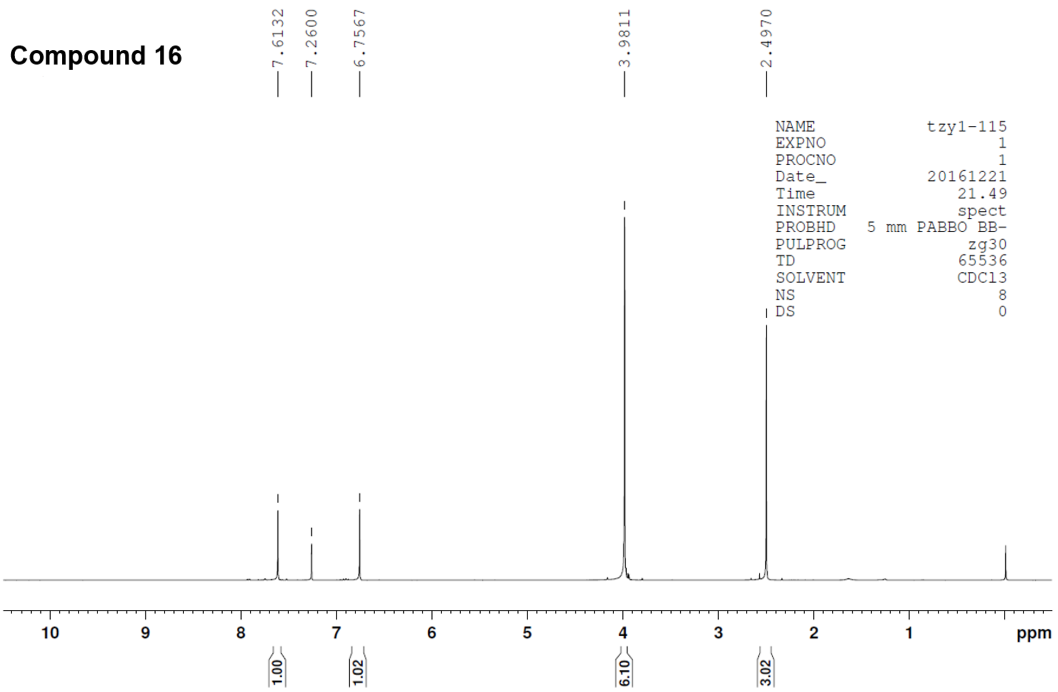

^{13}C NMR of compound **16**

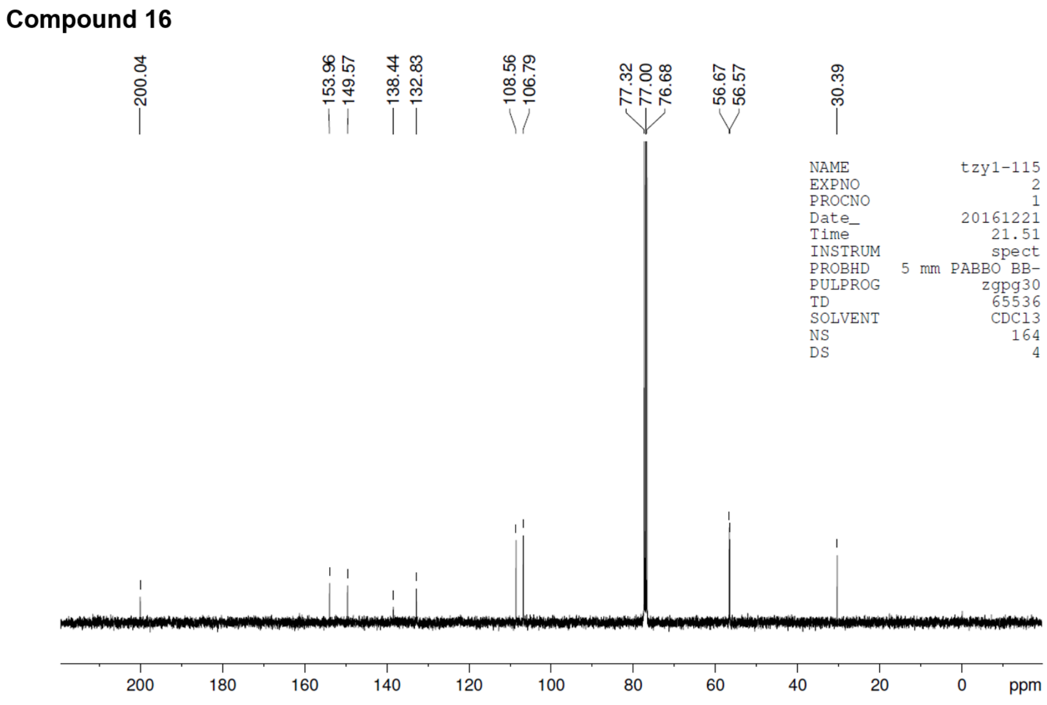

^{1}H NMR of compound **17**

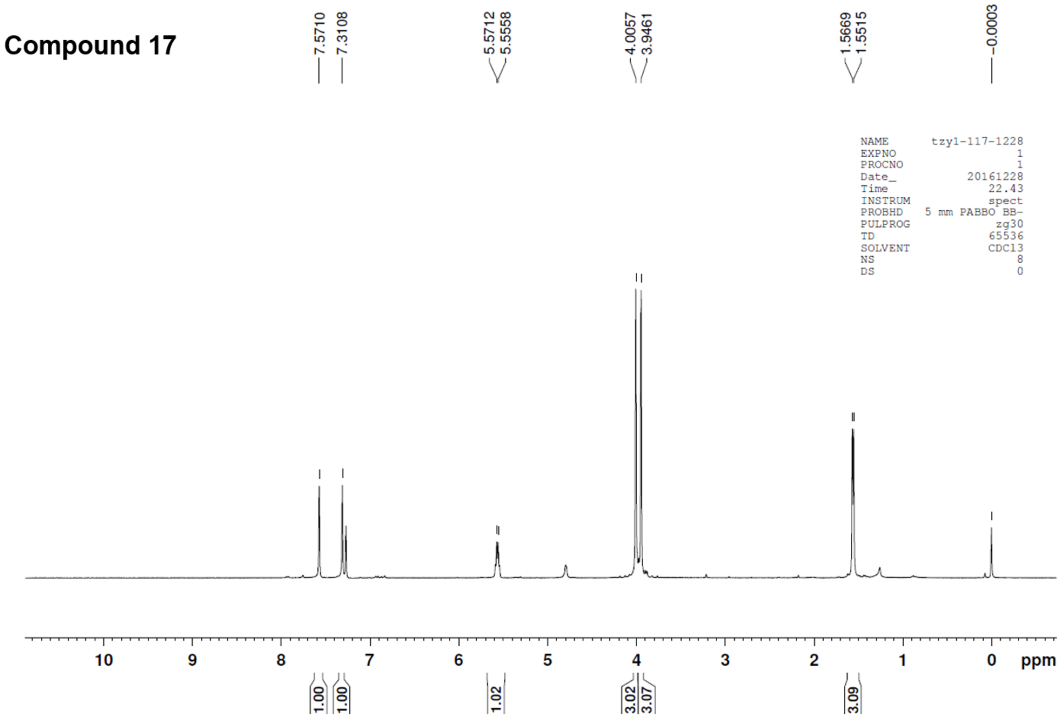

^{13}C NMR of compound **17**

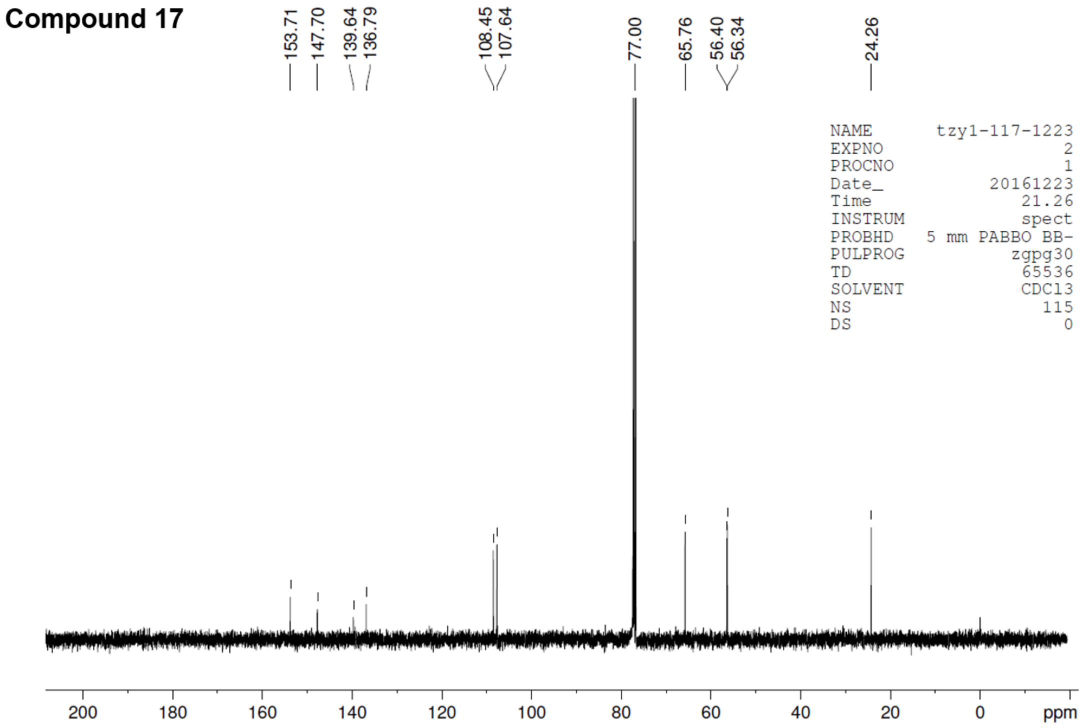

^{1}H NMR of compound **18**

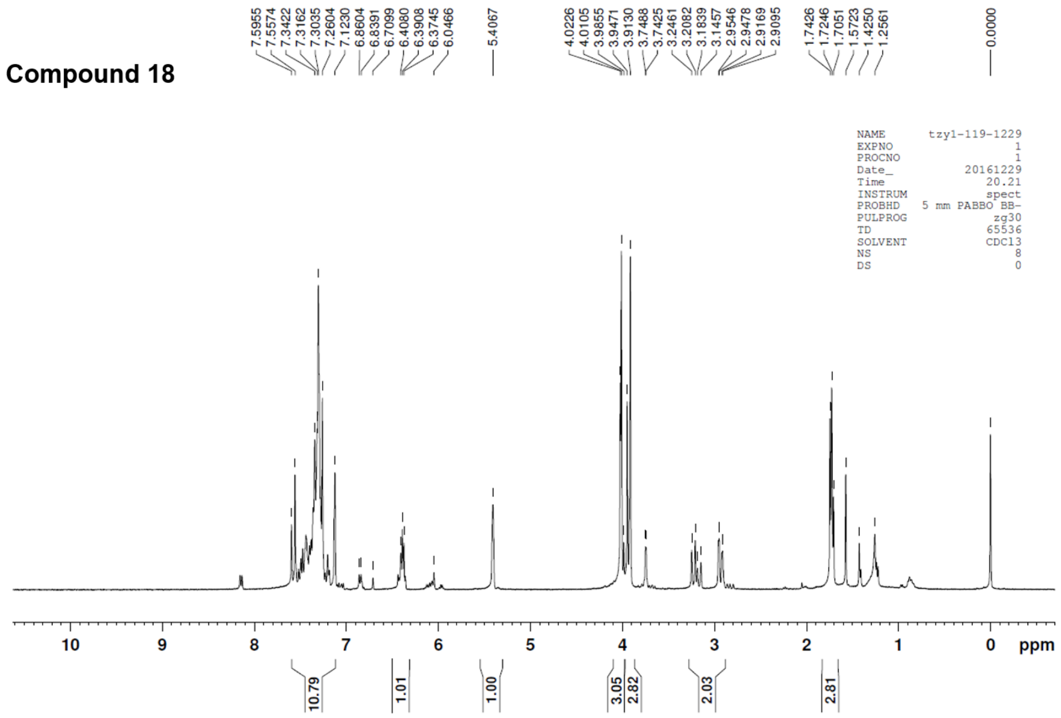

^{13}C NMR of compound **18**

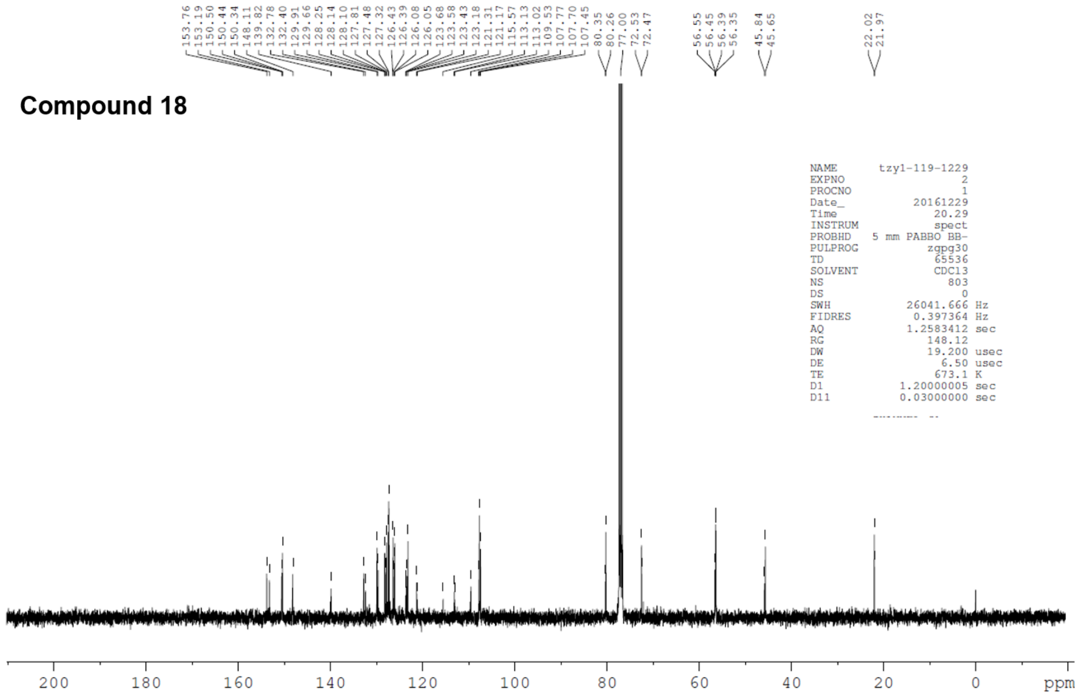

HRMS of compound **14**

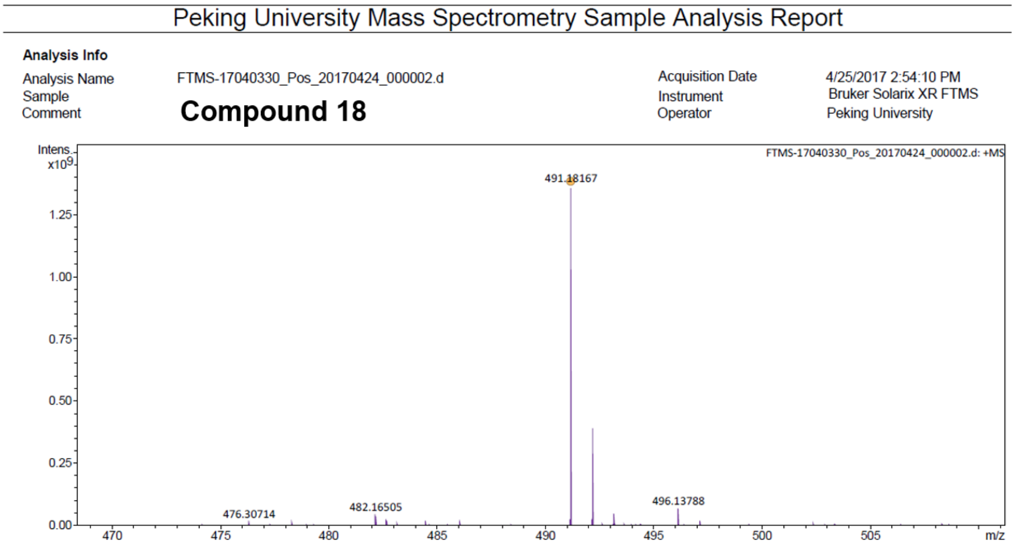

Meas. m/z	#	Ion Formula	Score	m/z	err [ppm]	Mean err [ppm]	mSigma	rdb	e⁻ Conf	N-Rule
491.181673	1	C27H27N2O7	100.00	491.181278	-0.8	-2.2	11.7	16.0	even	ok
